# Quantification of Silent Cerebral Infarction on High-Resolution FLAIR and Cognition in Sickle Cell Anemia

**DOI:** 10.3389/fneur.2022.867329

**Published:** 2022-06-29

**Authors:** Hanne Stotesbury, Jamie M. Kawadler, Jonathan D. Clayden, Dawn E. Saunders, Anna M. Hood, Melanie Koelbel, Sati Sahota, David C. Rees, Olu Wilkey, Mark Layton, Maria Pelidis, Baba P. D. Inusa, Jo Howard, Subarna Chakravorty, Chris A. Clark, Fenella J. Kirkham

**Affiliations:** ^1^Developmental Neurosciences, UCL Great Ormond Street Institute of Child Health, London, United Kingdom; ^2^Division of Psychology and Mental Health, Manchester Centre for Health Psychology, University of Manchester, Manchester, United Kingdom; ^3^King's College London, London, United Kingdom; ^4^North Middlesex University Hospital NHS Foundation Trust, London, United Kingdom; ^5^Haematology, Imperial College Healthcare NHS Foundation Trust, London, United Kingdom; ^6^Department of Haematology and Evelina Children's Hospital, Guy's and St Thomas' NHS Foundation Trust, London, United Kingdom; ^7^Clinical and Experimental Sciences, University of Southampton, Southampton, United Kingdom

**Keywords:** anemia, sickle cell, silent cerebral infarction, ischemia, white matter hyperintensities, magnetic resonance imaging, cognition, intelligence quotient

## Abstract

Research in sickle cell anemia (SCA) has used, with limited race-matched control data, binary categorization of patients according to the presence or absence of silent cerebral infarction (SCI). SCI have primarily been identified using low-resolution MRI, with radiological definitions varying in lesion length and the requirement for abnormality on both fluid attenuated inversion recovery (FLAIR) and T1-weighted images. We aimed to assess the effect of published SCI definitions on global, regional, and lobar lesion metrics and their value in predicting cognition. One hundred and six patients with SCA and 48 controls aged 8–30 years underwent 3T MRI with a high-resolution FLAIR sequence and Wechsler cognitive assessment. Prevalence, number, and volume of lesions were calculated using a semi-automated pipeline for SCI defined as: (1) Liberal: any length (L-SCI); (2) Traditional: >3 mm in greatest dimension (T-SCI); (3) Restrictive; >3 mm in greatest dimension with a corresponding T1-weighted hypo-intensity (R-SCI). Globally, as hypothesized, there were large effects of SCI definition on lesion metrics in patients and controls, with prevalence varying from 24–42% in patients, and 4–23% in controls. However, contrary to hypotheses, there was no effect of any global metric on cognition. Regionally, there was a consistent distribution of SCI in frontal and parietal deep and juxta-cortical regions across definitions and metrics in patients, but no consistent distribution in controls. Effects of regional SCI metrics on cognitive performance were of small magnitude; some were paradoxical. These findings expose the challenges associated with the widespread use of SCI presence as a biomarker of white-matter injury and cognitive dysfunction in cross-sectional high-resolution MRI studies in patients with SCA. The findings indicate that with high-resolution MRI: (1) radiological definitions have a large effect on resulting lesion groups, numbers, and volumes; (2) there is a non-negligible prevalence of lesions in young healthy controls; and (3) at the group-level, there is no cross-sectional association between global lesion metrics and general cognitive impairment irrespective of lesion definition and metric. With high-resolution multi-modal MRI, the dichotomy of presence or absence of SCI does not appear to be a sensitive biomarker for the detection of functionally significant pathology; the search for appropriate endpoints for clinical treatment trials should continue.

## Introduction

**Figure 1:**
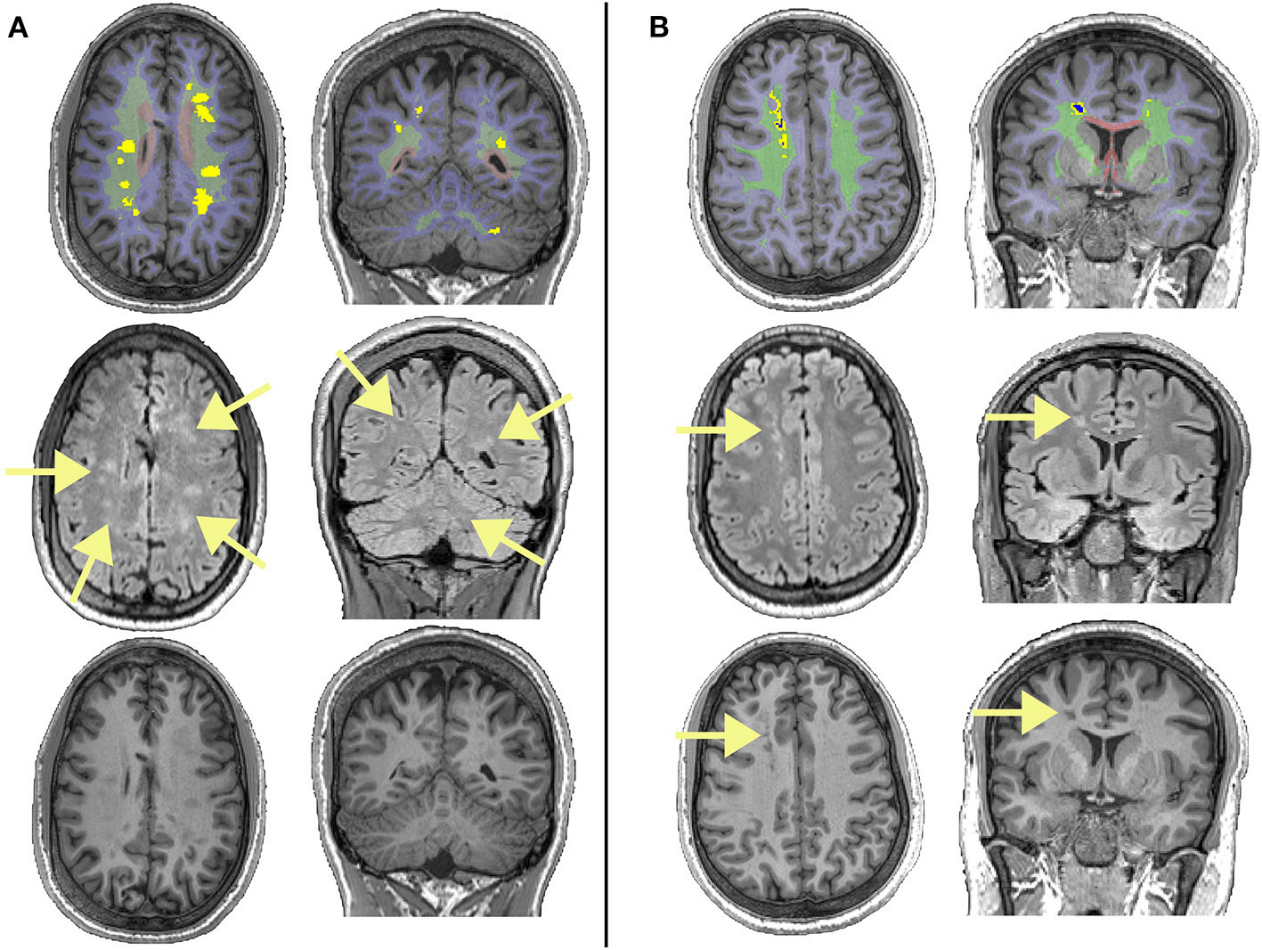
Examples of different lesions in two patients with sickle cell anemia. The top row shows regions of interest overlaid on T1-weighted images, with the juxta-cortical region shown in light blue, the deep region shown in light green, the periventricular region shown in light red, lesions that survived the FLAIR threshold (equation 1) shown in bright yellow, and lesions that survived the T1 threshold (equation 2) shown in dark blue. The middle row shows corresponding FLAIR images, with arrows showing the locations of lesions. The bottom row shows corresponding T1w images, with arrows showing the locations of lesions that survived the T1 threshold and restrictive definition. **(A)** Showing deep lesions meeting the liberal and traditional definitions in a male patient with sickle cell anemia aged 19. Despite having high FLAIR lesion burden for both the liberal and traditional definitions, this patient had relatively low lesion burden on T1, with none of the lesions shown meeting the restrictive definition. **(B)** Showing deep lesions meeting the liberal, traditional, and restrictive definitions in a male patient with sickle cell anemia aged 20. This patient had a high FLAIR lesion burden, with some voxels within lesion masks meeting the restrictive definition.

People with sickle cell anemia (SCA) are at risk of silent cerebral infarction (SCI) (1). Traditionally, SCI in children with SCA has been defined using the Silent Infarct Transfusion trial criteria: an area of abnormally high signal intensity on T2-weighted (T2-w) and/or fluid-attenuated-inversion-recovery (FLAIR) MRI (1.5T) measuring at least 3 mm in greatest dimension, visible on two planes, and with no corresponding focal deficit (i.e., traditional SCI definition, T-SCI; Figure 1) (2, 3). The majority of SCI are “deep” white matter hyperintensities (WMH), but “juxtacortical” and “periventricular” SCI have also been described (4, 5).

SCI occur in patients with SCA as young as 7 months (6), and are associated with future neurological complications, including new and enlarging SCI as well as ischemic stroke (7). Prevalence appears to increase throughout life, with SCI detected in 25% of patients by 6 years (5), 39% by 18 years (8), and 53% by 30 years (9). Using low-resolution sequences, presence of SCI has also frequently been associated with cognitive impairment across several domains (10), which may manifest as poor academic attainment (11). The American Society for Hematology (ASH) guidelines have therefore proposed that patients with SCA or HbSβ_0_ thalassemia should undergo MRI to detect SCI (12), while the use of presence of SCI as a biomarker for risk of cognitive difficulties was recently discussed at the Food and Drug Administration (FDA/ASH) meeting on endpoints for clinical trials in SCA (13). Findings have, however, been mixed, with several more recent studies failing to detect differences between patients with and without SCI in various cognitive domains, including general intelligence (IQ), processing speed and working memory (14–16).

Beyond the pediatric T-SCI definition, a more restrictive SCI definition (R-SCI) (Figure 1), based on studies in adults without SCA, was proposed in the first large-scale study of cognition in adults with SCA, requiring a hyperintensity of more than 5 mm in greatest dimension on T2w-MRI along with a corresponding hypo-intensity on T1-weighted (T1w) MRI (17). However, presence of R-SCI does not appear to predict general cognitive performance (i.e., IQ) in adults (17) or children (18) with SCA independently. Several studies employing a more liberal definition of SCI (e.g., hyperintensity of any length, L-SCI) have also failed to find associations with cognition (14, 19, 20).

Discrepancies in prevalence and association with cognition may relate to differences in the precise definition of SCI, clinical care, sample characteristics including age and cognitive range, and advances in technology (21). Studies using lower field strength magnets and lower-resolution sequences (e.g., 3–5 mm slice thicknesses with a slice gap) will by definition miss some lesions meeting liberal and traditional criteria, and there is evidence that lesion detectability increases with increasing magnet strength; in a study of adults with SCA, the prevalence of L-SCI increased from 60% at 3T (1 mm resolution) to 90% at 7T (0.8 mm resolution) (22).

There are relatively few data on the prevalence of SCI or any association with cognition in age- and race- matched controls and it is unclear whether the combination of increased lesion detectability and more liberal criteria improves or disrupts the ability to predict cognitive outcomes based on SCI presence alone in SCA. Greater detection of SCI in patients may include those with associated impairment, but smaller WMHs, also reported in children without SCA (23), and associated with healthy aging (24), may be less functionally significant.

In other populations where WMHs are common, including healthy older adults and those with dementia, there is typically no minimum lesion length requirement, and neurobehavioral correlates vary with lesion burden, regional classification, and lobar distribution (24, 25). Several recent studies employing higher resolution sequences in SCA populations have therefore considered one T-SCI per decade of life to be “normal,” and thus grouped participants according to whether they have an abnormal burden of T-SCI for age (15, 26). However, if the overall burden is important, L-SCI volume may plausibly serve as a more sensitive combination of lesion definition and metric. Whilst a few studies have considered relationships between global SCI volume and cognitive outcome in SCA (27–29), they all are based on relatively low resolution MRI sequences with thick slices (3–5 mm). Only one considered the potential impact of lesion distribution, albeit by grouping patients according to whether they had frontal (*n* = 7) or more widely distributed (*n* = 18) lesions (27).

Although the majority of clinical studies are now performed at 3T, and the global and regional burden of SCI can be quantified using semi-automated methods, no high-resolution imaging studies have investigated the effect of published SCI definitions, quantification metrics, and regional classification criteria on prediction of cognitive outcome in SCA. Determining the most sensitive combination of definition and metric using modern scanners is important, particularly as clinical trials have included or proposed including the appearance of new SCI as endpoints, partly on the basis that they are traditionally associated with cognitive impairment in SCA (13, 30, 31). In addition, an SCI endpoint may appear less subjective than a patient-reported outcome, and may also be more cost effective than full cognitive assessment requiring considerable time and effort for performance and interpretation. This was echoed in the aforementioned FDA meeting on endpoints, but the need for further validation using higher-resolution 3D sequences was also emphasized (13).

Therefore, the purpose of this study was to investigate, using previous radiological definitions, high-resolution FLAIR and semi-automated methods, the potential clinical utility of SCI as a biomarker of general cognition. Specifically, we hypothesized that there would be significant differences in global lesion metrics depending on the definition employed, with the most liberal definition (L-SCI) identifying significantly greater lesion burden. We also hypothesized that global lesion metrics based on the L-SCI definition would best predict general cognitive performance. In addition, we explored regional and lobar lesion metrics based on different SCI definitions and their value in predicting general cognition.

## Materials and Methods

### Sample

People with homozygous SCA (HbSS) or HbSß_0_-thalassemia were recruited to three on-going and concurrent studies at UCL Great Ormond Street Institute of Child Health between 2015 and 2019: the Sleep and Asthma Cohort follow-up (SAC) (32), the Prevention of Morbidity in Sickle Cell Anaemia baseline investigation (POMS) (31) and a study of sleep in SCA (33). Controls were siblings and race-matched peers (i.e., Black British) of patients recruited to SAC. Patients were ineligible for SAC and POMS study participation if they were receiving nocturnal respiratory support at the time of enrollment, participating in a clinical trial evaluating blood transfusion or oxygen therapy, or had chronic lung disease (other than asthma) or existing respiratory failure. Additional exclusion criteria for the POMS study were hospital admissions for acute sickle complications within 1 month of enrollment, more than 6 hospital admissions for acute sickle complications within 12 months of enrollment, overnight oximetry showing mean overnight saturation of <90% for more than 30% of total sleep time, severe sleep apnea defined by 4% oxygen desaturation index >15/h, and chronic blood transfusion or transfusion within 3 months of enrollment. For the SAC and sleep studies patients were enrolled without regard to past sickle- or sleep-related morbidity or transfusion status. West London NHS (SAC; 05/Q0408/42, 11/EM/0084, 15/LO/0347), Yorkshire NHS, (POMS; 15/YH/0213), and University College London (community recruitment 14475/001) research ethics committees provided ethical approval, and participants/parents provided written informed consent.

### Cognitive Measures

IQ was estimated using the two-subtest Wechsler Abbreviated Scale of Intelligence (WASI; POMS participants) (34), the Wechsler Intelligence Scale for Children (WISC-IV; SAC participants <16 years) (35), or the Wechsler Adult Intelligence Scale (WAIS-IV; SAC participants >16 years) (36). Subtests from the WISC-IV (POMS and SAC participants <16 years old) or WAIS-IV (POMS and SAC participants >16 years old) measuring working memory (Digit Span, Arithmetic) and processing speed (Coding, Symbol Search) were used to calculate composite indices (working memory index, WMI and processing speed index, PSI, respectively). Participants were classified as cognitively impaired if either their WMI, PSI, or IQ score fell below 70, corresponding to the 2nd percentile (i.e., 2 standard deviations below the normative mean). For 1 patient and 1 control with invalid performance on the coding subtest, and 1 control with invalid performance on the Symbol Search subtest, Cancellation subtest scores were used to calculate PSI. Participants were assessed as close to the date of MRI as possible, with 76% undergoing both on the same day, and all undergoing both within 4.5 months.

### Hematological Measures

Peripheral oxygen saturation (SpO_2_) was recorded at rest using a single fingertip pulse oximeter (Masimo Pronto) reading for at least 3 min on the day of MRI (SAC) or the closest trial visit (POMS). For patients with SCA, use of disease-modifying treatments (i.e., chronic blood transfusion, hydroxyurea) and blood counts were collected from medical records using the closest scheduled routine clinic visit to date of MRI.

### Socioeconomic Measures

Educational attainment, estimated from UK postcode using the English Indices of Deprivation (37) provided an index of socio-economic status. This measure has demonstrated sensitivity in SCA patients (16), and reflects educational attainment in local areas based on several indicators: (1) average scores for pupils in state-funded schools at ages 7–11 and 14–16 years, (2) absence from state-funded secondary schools, (3) the proportion of people staying on in education/training post 16 years, entry to higher education, and (4) proportion of working adults with no/low qualifications and language proficiency. Total scores are ranked from 1 to 10, with 1 representing the most deprived.

### Magnetic Resonance Imaging

MRI was performed on a 3T Siemens Prisma (Erlangen, Germany) with 80 mT/m gradients and a 64-channel receive head coil. The protocol included coronal high-resolution 3D FLAIR (TR = 5,000 ms, TE = 395 ms, voxel size = 0.65 × 0.65 × 1.0 mm, scan time = 6 min 22 s), axial 2D T2-w turbo spin echo (TR = 8 420 ms, TE = 68 ms, voxel size = 0.50 × 0.50 × 4.0 mm, scan time = 2 min, 50 s), T1-w magnetization-prepared rapid acquisition gradient echo (MPRAGE; TR = 2,300 ms, TE = 2.74 ms, TI = 909 ms, flip angle = 8°, voxel size = 1 × 1 × 1 mm, scan time = 5 min, 21 s) and 3D time-of-flight MRA sequences (TR = 21.0 ms, TE = 3.4 ms, scan time= 5 min, 33 s).

### SCI Definitions

**Figure 2:**
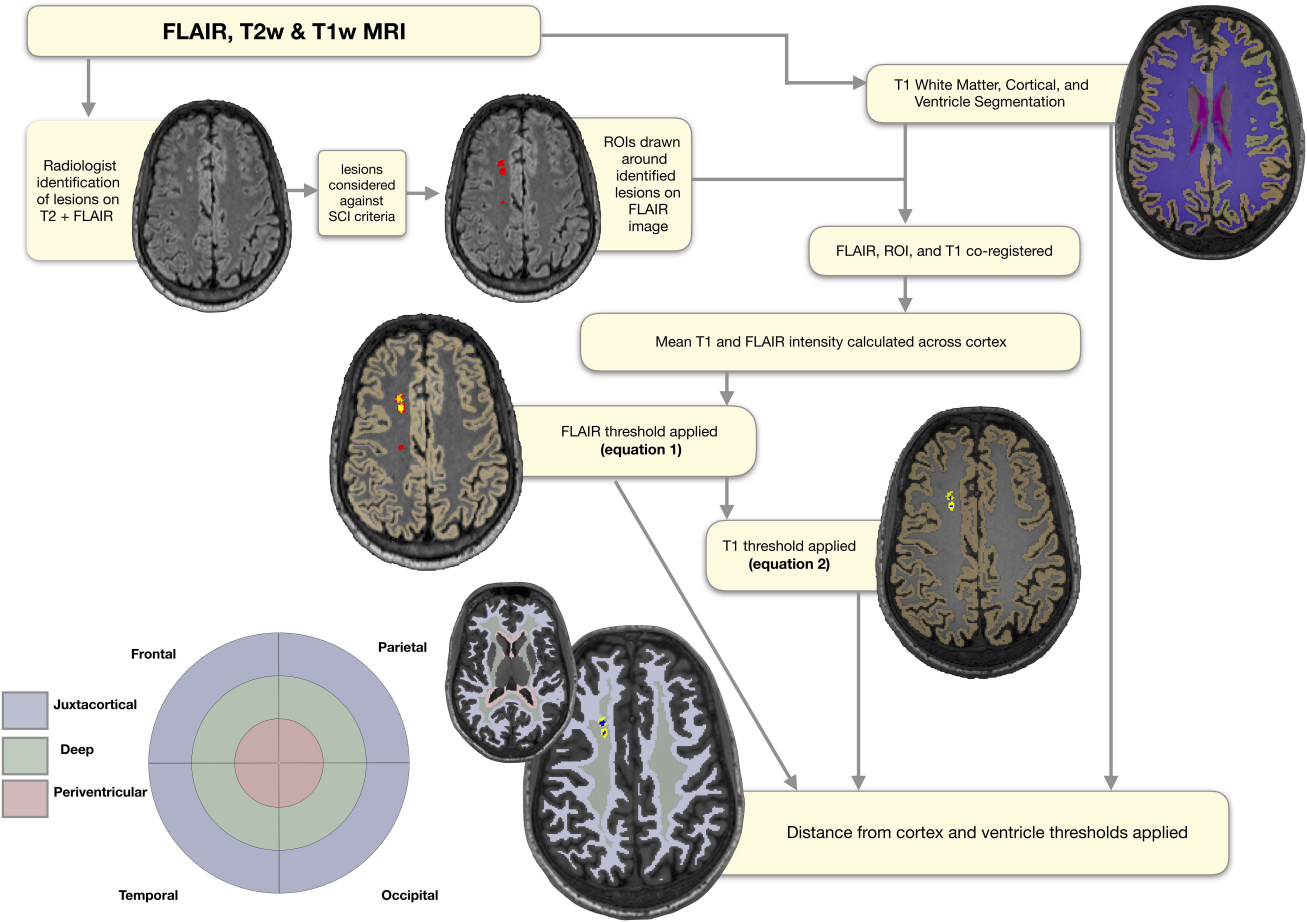
Semi-automated pipeline. Showing regions of interest (ROIs) at each stage of the semi-automated pipeline overlaid on a FLAIR image from a representative participant with sickle cell anemia (male, 25 years old). The initial ROIs drawn around the lesions identified by the neuroradiologist are shown in red. The ROIs following application of the FLAIR threshold are shown in yellow, and the ROIs following application of the T1 threshold in blue. The masks used for regional classification, along with the corresponding simplified bullseye plot, are shown in light blue for the juxta-cortical region, light green for the deep region, and light pink for the periventricular region.

The full pipeline for definition, quantification, and classification of SCI in participants without focal neurological symptoms is shown in Figure 2. First, an experienced pediatric neuroradiologist, blind to disease status, identified regions of abnormally high signal intensity indicating an ischemic lesion and recorded the lobe in which lesions were located. Lesions were identified on the coronal FLAIR and confirmed on the axial T2-weighted image, i.e., visible in two planes as lesions in accordance with the silent infarct transfusion (SIT) trial protocol (2). Clearly distinguishable SCI “mimics” were excluded (e.g., linear perivascular spaces running in the direction of small veins). Using Horos (https://horosproject.org), lesions were then manually measured on the coronal FLAIR, counted, and considered against three definitions;

(1) L-SCI: any length (19, 29).
(2) T-SCI*:* at least 3 mm in greatest dimension (i.e., SIT-trial definition) (2).
(3) R-SCI*:* at least 3 mm in greatest dimension with a corresponding hypo-intensity on T1w MRI (17).

### Quantification of Global SCI

To extract participant-specific estimates of total intracranial volume, as well as masks of the cortex, white matter, and ventricles (lateral and fourth), cortical reconstruction and volumetric segmentation were performed on T1-w images using Freesurfer (Center for Biomedical Imaging, Massachusetts, USA; http://surfer.nmr.mgh.harvard.edu/). Regions of interest (ROIs) were then manually drawn around the identified lesions on native FLAIR images, before FLAIR and T1w images were bias-corrected using SPM (Wellcome Trust Centre for Neuroimaging, London, UK; http://www.fil.ion.ucl.ac.uk/spm), and linearly affine-aligned using FSL (FMRIB, Oxford, UK; https://fsl.fmrib.ox.ac.uk/fsl/fslwiki/FSL). Based on a previously published SCA-specific pipeline (29), for each participant the mean FLAIR intensity across cortex was calculated, and the following lower threshold was used to determine which voxels within ROIs should be included in a total FLAIR lesion burden mask (Figure 2);


(1) $$\begin{array}{c} {\text{LowerThr}}_{flair}\;=\;1.02\;\times \;{\text{mean}}_{\text{FLAIR}cortex} \end{array}$$

Next, the FSL cluster tool separated the total FLAIR lesion mask into individual FLAIR lesion masks that corresponded with each lesion initially identified by the neuroradiologist. For consistency with the FLAIR hyperintensity definition, and to operationalize and automate the visual definition of a corresponding T1 hypo-intensity provided by Choudhury et al. (18) (i.e., that lesions appear either iso- or hypo- intense to surrounding gray matter), the mean cortical T1 intensity for each participant was calculated. The following upper threshold was then used to determine which, if any, voxels within individual FLAIR lesion masks should be included in a T1-thresholded FLAIR lesion cluster mask (Figure 2):


(2) $$\begin{array}{c} {\text{UpperThr}}_{t1}\;=\;1.02\;\times {\text{ mean}}_{\text{T1}cortex} \end{array}$$

The total number and volume of lesions surviving the semi-automated pipeline were then calculated for lesions that met the L-SCI, T-SCI, and R-SCI definitions.

### Classification and Quantification of Regional SCI

To classify lesions as periventricular, deep, or juxtacortical, FSL was used to create participant-specific maps quantifying distances from the ventricle and cortex masks from Freesurfer. Although established absolute distance rules for classification of lesions exist from work with adult non-SCA samples, we elected not to apply these given the younger age range of our sample. Instead, for each participant's “distance from ventricle” map, an upper threshold corresponding to the 5th percentile of voxels in white matter was used to create a periventricular region mask (Figure 2). Voxels within the periventricular region mask were then excluded from the participant-specific cortex distance map to avoid overlap. Next, an upper threshold corresponding to the 75th percentile of voxels in white matter was applied to the cortex distance map to create a juxtacortical region mask. Remaining white matter voxels were then used to generate a deep white matter region mask. These distance thresholds were chosen as they gave the most accurate classification compared with neuroradiologist classification for a random subsample of patients (*N* = 10, pipeline accurately classified lesions in all cases). We had also experimented with the 10th percentile of white matter for the distance from ventricles mask, and the 85th percentile of voxels for the distance from cortex mask. For the 10th percentile periventricular threshold, 1 lesion that was classed as periventricular by the neuroradiologist fell in the deep regional mask. Similarly, for the 85th percentile juxta-cortical threshold, 1 lesion that was classed as deep by the neuroradiologist fell in the juxta-cortical mask.

The number and volume of lesions of each class were then quantified. For the number of lesions, in the event of overlap between FLAIR lesion masks and periventricular, juxtacortical, and/or deep region masks, lesions were classified according to whichever regional mask contained the greatest number of FLAIR lesion voxels (Figure 1). If regions contained an equal number of lesion voxels, the neuroradiologist was consulted for classification (*N* = 3 cases). For the volume of lesions, the number of voxels within each regional mask was used, allowing for within-lesion overlap between regions. A simplified “bulls-eye” representation of lesion burden (38) in 12 regional-lobar zones was created (Figure 2), with the lobe as designated by the neuroradiologist.

### Statistical Analysis

Analyses were performed in RStudio Desktop 1.0.153 using packages including “companion to applied regression” (39) and “global validation of linear models” (40). Prior to statistical analyses, variables were assessed for normality and equality of variance. Non-normal lesion metrics (i.e., number and volume) were log-transformed. Estimates of global lesion metrics (i.e., total prevalence, number, and volume) were compared between L-SCI, T-SCI, and R-SCI definitions using Cochran's-Q and Friedman tests, and between patients and controls using Chi-Squared, Fishers Exact (*N* < 10), and Mann-Whitney U tests. Effect sizes were estimated using maximum-corrected measure of effect size ($\eta_{\text{Q}}^{2}$), Kendall's W(w), Cramér's-phi (ϕ*)*, Cohens *d* (*d*), and correlation coefficients (*r*), and interpreted using Cohen's conventions (small d = 0.2, $\eta_{\text{Q}}^{2}$/w/ϕ/r = 0.1, medium d = 0.5, $\eta_{\text{Q}}^{2}$*/w/*ϕ*/r/* = 0.3, large d = 0.8, $\eta_{\text{Q}}^{2}$ *w/*ϕ*/r* = 0.5) (41).

### Global Models

For each SCI definition, regression models estimating IQ, WMI, PSI, and cognitive impairment from an increasingly complex combination of global lesion metrics were computed (Table 1). Models including binary indicators denoting the presence of SCI or the presence of at least 1 SCI that met the relevant definition per decade of life (Table 1; Model 1) were compared to models where binary indicators were included alongside continuous quantifiers, representing either the global number (Table 1; Model 3) or volume (Table 1; Model 5) of SCI according to the relevant definition.

**Table 1:** Representation of lesion metrics included in regression models.

***Model***	**Lesion metrics included as predictors**
1.	SCI_y/n_
2.	SCI_y/n_ + SCI_y/n_^*^ SCA_y/n_
3.	SCI_y/n_ + SCI_number_
4.	SCI_y/n_ + SCI_y/n_ ^*^ SCA_y/n_ + SCI_number_ + SCI_number_ ^*^ SCA_y/n_
5.	SCI_y/n_ + SCI_volume_
6.	SCI_y/n_ + SCI_y/n_^*^ SCA_y/n_ + SCI_volume_ + SCI_volume_^*^ SCA_y/n_

*+ = addition*;

^*^ = multiplication.

*SCI_y/n_, binary variable representing presence or absence of silent cerebral infarction; SCI_number_, continuous variable representing the number of lesions; SCI_volume_, continuous variable representing the volume of lesions; SCA_y/n_, binary variable representing presence or absence of sickle cell anemia*.

To explore potential effects of SCA status on the effect of SCI metric, where distributions allowed, models were also computed with interaction terms composed of [mean-centered SCA status] X [lesion metric] (Table 1; Models 2, 4, and 6). Models were also re-computed in a subset including SCA patients only.

### Regional Models

As for the global lesion models, exploratory regional models including binary indicators denoting regional (i.e., deep, juxta-cortical and periventricular) or lobar (i.e., frontal, parietal, temporal, and occipital) lesion presence, were compared to corresponding models including indicator variables alongside continuous quantifiers denoting SCI number or volume. Models were also computed with a predictor based on the total number of regional-lobar zones with lesion voxels. Previous reports indicate a significantly unequal regional and lobar distribution of lesions in patients with SCA (4). Therefore, we expected a bimodal distribution of relatively commonly affected regions/lobes vs. relatively rarely affected regions/lobes. To avoid large between-region/lobe differences in statistical power and statistical inferences based on very small numbers of participants, only the most commonly affected regions and lobes were included in regional models.

### Model Statistics

Across all models, a probability threshold of *p* < 0.05 was considered statistically significant, and model assumptions and variance inflation factors (VIF) were assessed. If there was significant multi-collinearity (i.e., VIF > 5) between binary indicator or continuous quantifier variables, binary indicator variables were dropped from models. The predictive value of definitions was assessed by comparing beta-values and semi-partial correlation coefficients for any significant lesion predictors between the models. Based on prior literature (16, 18, 42), pre-selected covariates comprising age, sex, total intracranial volume, SpO_2_, and education deciles, were included in all models. In models re-computed in the SCA patient subset, hemoglobin was additionally included as a covariate. Prior to their inclusion in models, exploratory correlation analyses were performed between variables. Correlations for comparison of the number and volume of T-SCI, L-SCI, and R-SCI with FSIQ, WMI and PSI were additionally computed separately for children ≤15.99 years and adults >16 years. The relevant group means were imputed for four patients with missing hemoglobin and seven controls with missing SpO_2_. To match prior research (18), and maximize our ability to detect effects of SCI should they exist, we did not correct for multiple comparisons.

## Results

### Participants

**Figure 3:**
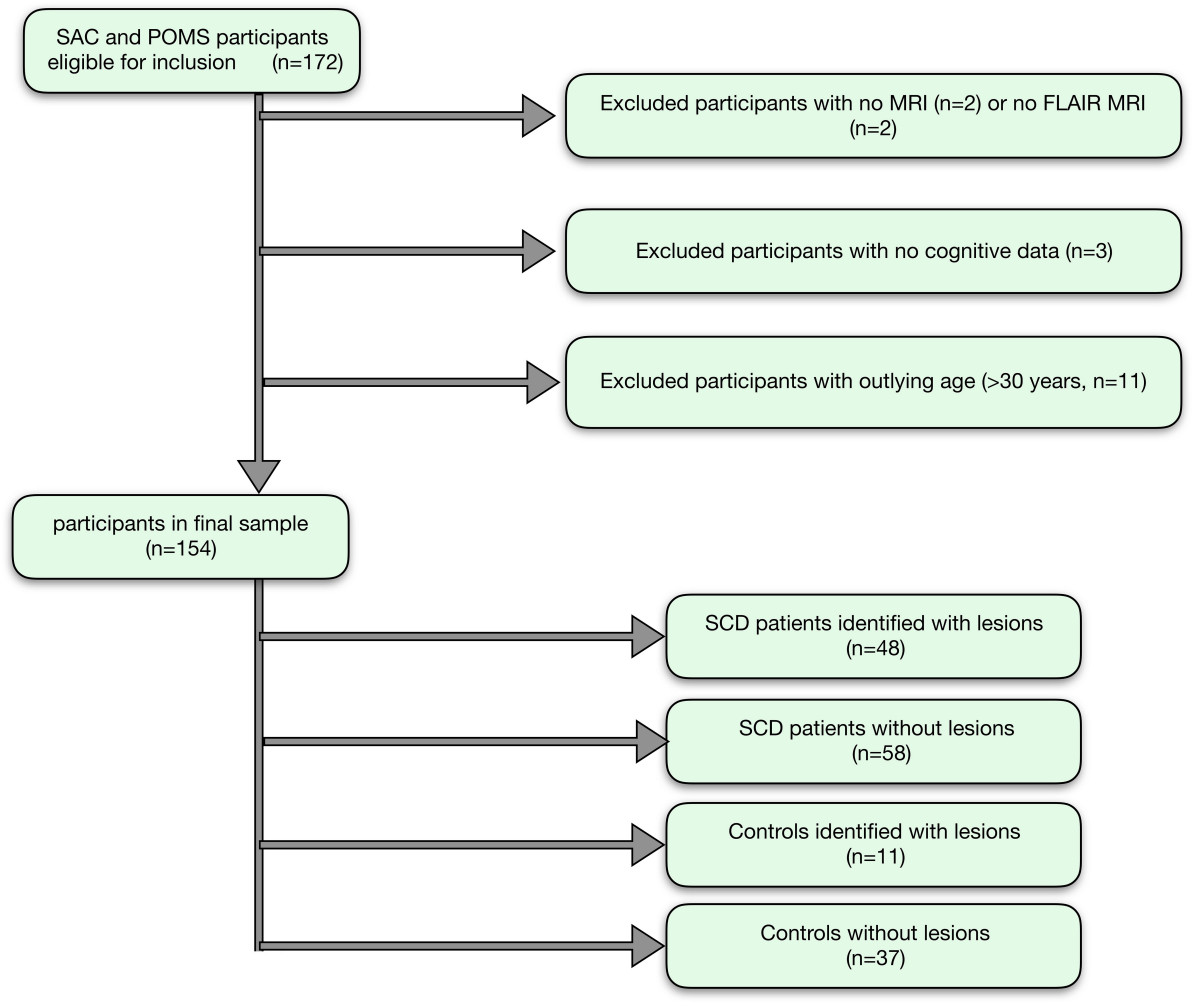
Participant flow-chart. Showing the flow of participants, with reasons for exclusion, and the final groups for analysis.

Of 172 recruited participants, 106 patients with SCA (53 male, age range 8–29 years, 105 HbSS, 1 HbSß_0_-thalassemia) and 48 race- and age- matched controls (20 male, age range 8–30 years; 27 HbAA, 19 HbAS, 2 HbAC; 31 siblings of patients) were included in the final sample (Figure 3).

Two patients had large vessel vasculopathy, six were receiving chronic transfusion, 16 had been transfused within 3 months, and 42 were prescribed hydroxyurea. SpO_2_ was significantly lower in patients than controls, and hemoglobin was lower than normative values, but there were no significant differences in age, sex, or education deciles (Table 2).

**Table 2:** Sample demographics and cognitive performance.

	**SCA (*n* = 106)**	**Control (*n* = 48)**	***Between-group differences***
***Demographic variables***	**Count (percentage)**		
Sex	53 Male (50.00%)	20 Male (41.67%)	*Chi-Squared test* ${\text{X}}_{1}^{2}$ = 0.62, *p* = 0.43, *ϕ =* 0.06
	Median (range)		*Mann-Whitney U test*
Age (yr)	16.56 (8–29)	16.55 (8–30)	U = 2,594.5, *p* = 0.85, 95% CI = −1.64–1.98, *r* = 0.02
Education Decile	5.00 (1–10)	5.00 (2–10)	U = 2,353.5, *p* = 0.45, 95% CI = −0.99–0.00, *r* = 0.06
***Clinical variables***	**Count (percentage)**		***Mann-Whitney U test***
Chronic Transfusion	6 (5.66%)	–	–
Acute Transfusion <3 months	16 (15.10%)	–	
Hydroxyurea	42 (39.62%)	–	
Hemoglobin (g/dl)	87.70 (60–134)	–	
SpO_2_ (%)	97.00 (89–100)	99.00 (93–100)	U = 3,742.5, *p < * 0.005^*^, 95% CI = 1.00–2.00, r = 0.38
***Cognitive variables***	**Mean (SD)**		***Students t-test***
Intelligence quotient (IQ)	93.15 (13.28)	*All* 97.29 (11.78) *HbAA/HbAC 98.31 (10.52)* *HbAS 95.74 (13.64)*	*t*_(101.6)_ = 1.94, *p =* 0.06^∧^, 95% CI = −0.09–8.37, d = 0.33 *t_(31.7)_ = 0.70, p = 0.49, 95% CI = −4.94–10.10, d = 0.21*
Working memory index (WMI)	92.04 (14.18)	*All* 98.90 (13.34) *HbAA/HbAC 99.03 (13.54)* *HbAS 98.68 (13.40)*	*t*_(96.1)_ = 2.90, *p < * 0.005^*^, 95% CI = 2.16–11.56, d = 0.50 *t_(38.9)_ = 0.09, p = 0.93, 95% CI = −7.68–8.38, d = 0.03*
Processing speed index (PSI)	89.56 (12.99)	*All* 97.10 (13.03) *HbAA/HbAC 97.62 (13.24)* *HbAS 96.32 (13.01)*	t_(90.6)_ = 3.33, *p < * 0.005^*^, 95% CI = 3.05–12.05, d = 0.58 *t_(39.1)_ = 0.33, p = 0.74, 95% CI = −6.52–9.13, d = 0.74*
	**Count (percentage)**		***Fishers Exact test***
Cognitive impairment (CI)	12 (11.32%)	*HbAA* 1 (2.08%) *HbAC 0 (0.00%)* *HbAS 1 (5.26%)*	OR = 5.95, *p =* 0.06^∧^, 95% CI = 0.83–261.56 –

*Values are summary and test statistics. For between group-differences in cognitive variables, the top row compares patients and controls, while the second row compares controls with and without sickle cell trait*.

∧ *p < 0.1*;

* *p < 0.05*.

*SCA, sickle cell anemia; HbAA/HbAC, control with hemoglobin A; HbAs, control with hemoglobin S, sickle cell trait; SpO_2_, peripheral oxygen saturation; SD, standard deviation; 95% CI, 95% confidence interval*.

### Cognitive Performance

**Figure 4:**
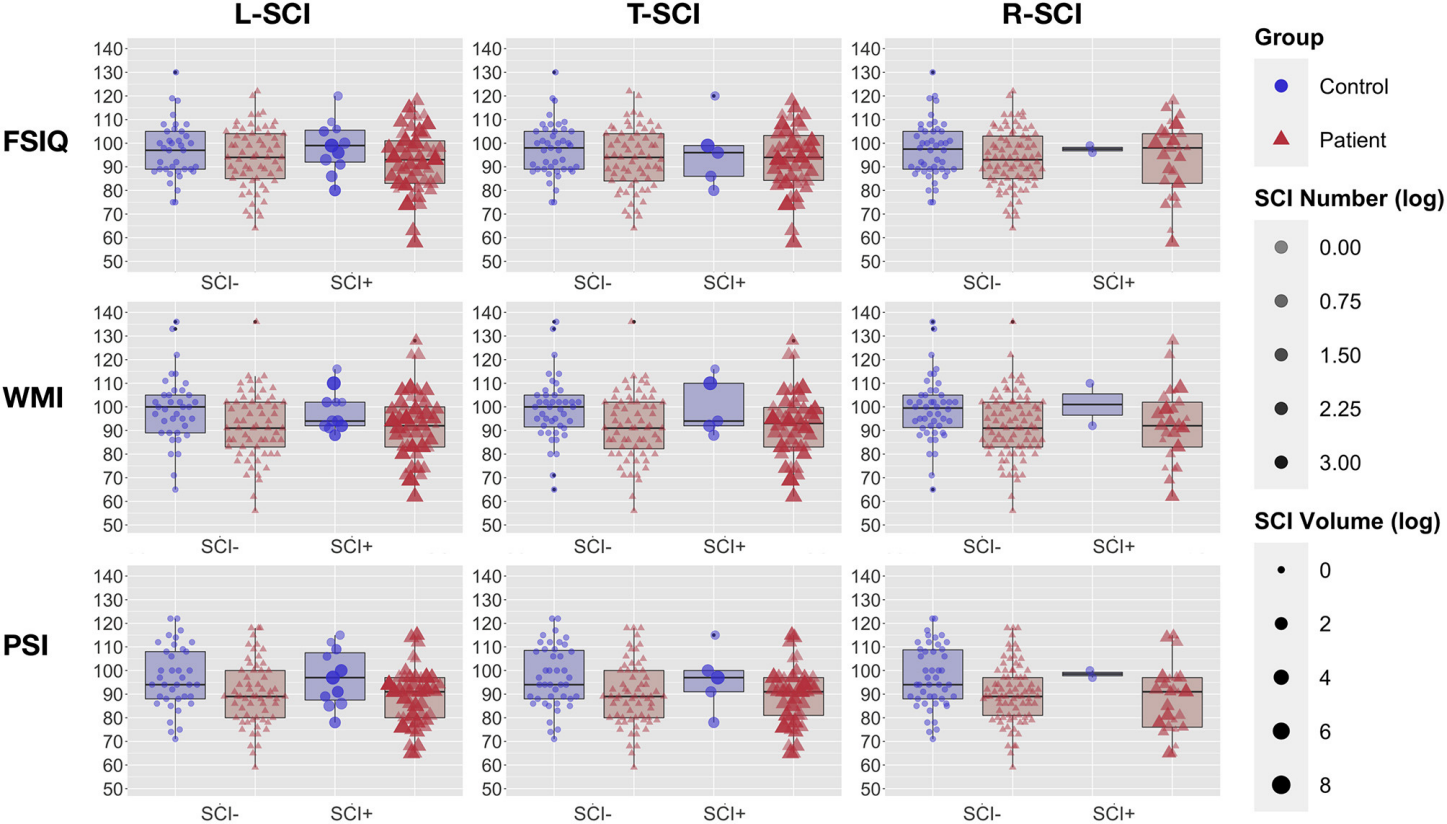
Cognitive Performance and Global Lesion Presence. Showing full-scale IQ (FSIQ: top row), working memory index (WMI: middle row), and processing speed index (PSI: bottom row) in patients with sickle cell anemia (red triangles) and controls (blue circles). Participants are grouped by silent cerebral infarct status (SCI+ = with silent cerebral infarction, SCI– without silent cerebral infarction) for the liberal definition (left column), traditional definition (middle column), and restrictive definition (right column). For each definition, point intensity is weighted by the log-transformed number of lesions, and point size by the log transformed volume of lesion.

Analyses that assessed differences in cognitive performance between patients with SCA and controls found that patients had lower scores of 7 WMI points, 8 PSI points (*ps* < 0.005), and 4 IQ points (*p* = 0.06, Table 2; Figure 4) than controls. Effect sizes for these cognitive differences were moderate to large. For controls, there were no significant differences in cognition between those with and without sickle cell trait (all *ps* > 0.5). 11.3% of people with SCA (12/106) were identified as having cognitive impairment (i.e., WMI, PSI, or IQ score < 70) compared with 4.2% (2/48) of controls (see Table 2). As shown in Figure 4, the raw data demonstrated greater variation in cognitive performance within than between lesion groups, with no clear dose-dependent effect of the total lesion number or volume, in patients or controls.

### Global SCI Metrics

Forty-five patients (42%, 23 male, median age = 16.9, range = 8–27 years) and 11 controls (23%, 2 male, median age = 14.8, range = 9–21 years, HbAS = 7) had lesions that met the L-SCI definition and survived the semi-automated pipeline. Cochran's Q tests revealed significant effects of SCI definition on lesion prevalence in both patients and controls; the L-SCI definition identified three more patients (7%) and six more controls (120%) with lesions compared to the T-SCI definition, and 20 more patients (80%) and nine more controls (450%) compared to the R-SCI definition (*ps* < 0.05; Table 3). A similar pattern was observed for the additional requirement that at least 1 lesion be allowed per decade of life and the median number and volume of lesions. Significant effects of SCA status on global lesion metrics were also observed, with a greater global prevalence, number, and volume of lesions observed in patients, irrespective of the SCI definition employed (*ps* < 0.005; Table 3).

**Table 3:** Global lesion characteristics.

	**L-SCI**		**T-SCI**		**R-SCI**		
	**SCA**	**HC**	**SCA**	**HC**	**SCA**	**HC**	***Within-group differences***
**Lesion prevalence**							
Total number of participants with lesions (% of total group)	45 (42.45%)	11 (22.92%)	42 (39.62%)	5 (10.42%)	25 (23.58%)	2 (4.17%)	*Cochrans Q test* SCA: Q*_2_* = 34.90, *p < * 0.005^*^, $\eta_{\text{Q}}^{2}$ = 0.11 HC: Q*_2_* = 14.00, *p < * 0.005^*^, $\eta_{\text{Q}}^{2}$ = 0.15
***Between-group differences***	*Chi-squared test* $X_{1}^{2}$ = 4.64, *p =* 0.031^*^, ϕ = 0.17		*Chi-squared test* $X_{1}^{2}$ = 11.90, *p < * 0.005^*^, ϕ = 0.28		*Fishers Exact test* OR = 7.03, *p < * 0.005^*^		
Total number of participants with >1 lesion per decade of life (% of total group)	37 (34.91%)	9 (18.75%)	32 (30.19%)	5 (10.42%)	12 (11.32%)	0 (0.00%)	SCA: Q*_2_* = 42.00, *p < * 0.005^*^, $\eta_{\text{Q}}^{2}$ = 0.14 HC: Q_2_ = 13.56, *p < * 0.005^*^, $\eta_{\text{Q}}^{2}$ = 0.14
***Between-group differences***	*Chi-squared test* $X_{1}^{2}$ = 3.38, *p =* 0.07^∧^, ϕ = 0.15		*Chi-squared test* $X_{1}^{2}$ = 6.03, *p =* 0.01^*^, ϕ = 0.20		*Fishers Exact test* OR = NA, *p =* 0.02^*^		
**Lesion number**							*Friedman test*
Median total number of lesions in participants with lesion meeting definition (range)	4 (1–29)	3 (1–12)	3 (1–26)	2 (1–8)	2 (1–10)	1 (1–1)	SCA: Q*_2_* = 76.80, *p < * 0.005^*^, W = 0.36 HC: Q*_2_* = 19.50, *p < * 0.005^*^, W = 0.20
***Between-group differences***	*Mann-Whitney U test* U = 2,000.00, *p =* 0.01^*^, r = −0.18		*Mann-Whitney U test* U = 1,781.00, *p < * 0.005^*^, r = −0.28		*Mann-Whitney U test* U = 2,036.00, *p < * 0.005^*^, r = −0.22		
**Lesion volume**							
							*Friedman test*
Median total lesion burden (volume, mm^3^) in participants with any lesion burden (range)	75 (1–5177)	22 (2–1282)	63.5 (1–5177)	19 (3–1075)	4 (1–268)	2 (2–2)	SCA: Q*_2_* = 81.10, *p < * 0.005^*^, W = 0.38 HC: Q*_2_* = 19.50, *p < * 0.005^*^, W = 0.20
***Between-group differences***	*Mann-Whitney U test* U = 1,938.00, *p < * 0.005^*^, r = −0.20		*Mann-Whitney U test* U = 1,774.00, *p < * 0.005^*^, r = −0.28		*Mann-Whitney U test* U = 2,036.00, *p < * 0.005^*^, r = −0.22		

*Values are summary and test statistics comparing lesion metrics between patients and controls groups (columns; Chi-Squared and Mann-whitney U tests) and within groups as a function of SCI definition (rows; Cochran's-Q and Friedman tests) ^∧^p < 0.1*;

* *p < 0.05. L-/T-/R-SCI, Liberal/Traditional/Restrictive silent cerebral infarction definitions; HC, healthy control; SCA, sickle cell anemia*.

T-SCI were detected in controls with sickle cell trait as young as 9 years of age, and in those with HbAA/AC as young as 12 years of age. A non-significantly greater proportion of controls with HbAS were identified as having SCI (L-SCI HbAA/AC: 4/29 [14%, 0 AC] vs. HbAS: 7/19 [37%]), irrespective of the definition (*ps* > 0.05).

### Global SCI Metrics and Cognition

**Figure 5:**
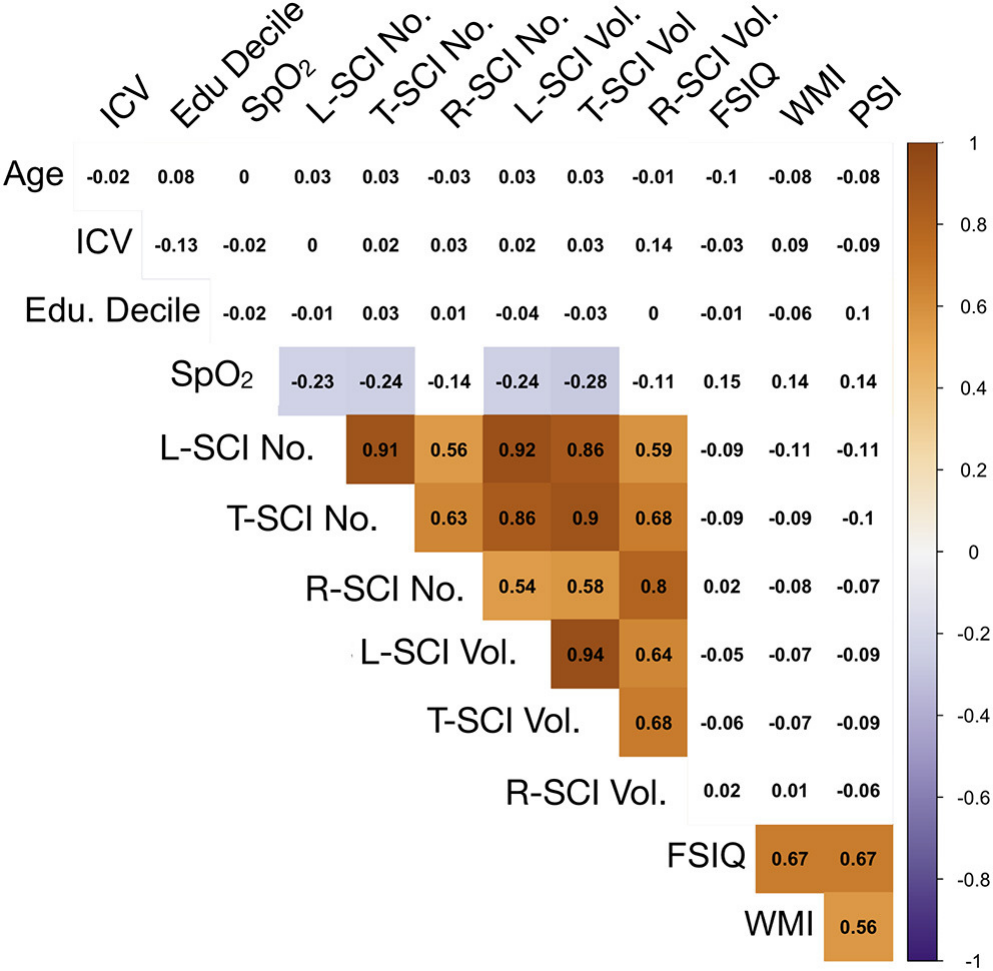
Correlations between model predictors. Showing relationships between continuous variables included in the global regression models. Values are zero-order Spearman's rank correlation coefficients. Shaded areas represent significant relationships (i.e., *p* < 0.05), with blue used to represent negative relationships, and orange used to represent positive relationships, and color intensity used to represent the strength of the relationships. ICV, intra-cranial volume; Edu. Decile, education decile; SpO_2_, peripheral oxygen saturation; SCI Lib, SCI liberal definition; SCI Trad., SCI traditional definition; SCI Rest, SCI Restrictive definition; No., Number; Vol, Volume; FSIQ, full-scale IQ; WMI, working memory index; PSI, processing speed index.

Exploratory Spearman's rank correlations revealed several significant relationships (Figure 5, Supplementary Figure 1). In addition to expected associations between closely related variables, decreases in SpO2 were modestly associated with increases in L-SCI and T-SCI lesion number and volume (rs = −0.23 to −0.28, ps < 0.05; Figure 5). When these analyses were conducted in only the SCA subsample, these associations remained significant, and other modest associations between decreases in hemoglobin and decreases in IQ (r = 0.29, *p* < 0.05) and PSI (r = 0.20, *p* < 0.05) were observed (Supplementary Figure 1). No univariate relationships were observed between lesion variables and any measure of cognition, and there were no relationships with total intracranial volume, age, or education deciles. When the SCA subsample was further stratified by age, there were no correlations for the L-SCI or T-SCI definitions, but volume and number of R-SCI were paradoxically positively correlated with IQ in children with SCA aged <16 years (Table 4). There were no correlations between volume or number of T-SCI, L-SCI or R-SCI and IQ in adults, nor any correlations with WMI or PSI in either age group (Table 4).

**Table 4:** Spearman correlations for comparison of cognitive outcomes and silent cerebral infarct volume and number using the Liberal (L-SCI), Traditional (T-SCI) and Restrictive (R-SCI) definitions.

	**Children (<16 y) with sickle cell anemia**						**Adults (≥16 y) with sickle cell anemia**					
	***N*** **=** **48**						***N*** **=** **57**					
	**IQ**		**WMI**		**PSI**		**IQ**		**WMI**		**PSI**	
**Silent cerebral infarct definition**	**r**	***p***	**r**	***p***	**r**	***p***	**r**	***p***	**r**	***p***	**r**	***p***
L-SCI total volume	0.172	0.2	0.006	0.9	−0.064	0.7	−0.191	0.2	−0.007	0.9	0.015	0.9
T-SCI total volume	0.182	0.2	−0.022	0.9	−0.040	0.8	−0.156	0.2	0.029	0.8	0.027	0.8
R-SCI total volume	0.308	0.03^*^	0.090	0.5	−0.093	0.5	−0.115	0.4	0.017	0.9	−0.024	0.9
L-SCI number	0.162	0.3	−0.007	0.9	−0.064	0.2	−0.204	0.1	0.012	0.9	0.019	0.9
T-SCI number	0.177	0.2	−0.029	0.8	−0.025	0.9	−0.159	0.2	0.061	0.7	0.038	0.8
R-SCI number	0.309	0.03^*^	0.088	0.6	−0.099	0.5	−0.152	0.3	−0.038	0.8	−0.048	0.7

* *p < 0.05*.

*y, years; IQ, intelligence quotient; WMI, working memory index; PSI, processing speed index; L-/T-/R-SCI, Liberal/Traditional/Restrictive silent cerebral infarction definitions*.

The results from the univariate correlation analyses were echoed in regression models estimating IQ, WMI, PSI, and cognitive impairment from global lesion metrics, where no significant effects emerged (Table A1), irrespective of the definition employed, the inclusion of interaction terms for patient status, the model complexity, or whether analyses were run across the whole sample or in people with SCA only.

### Regional SCI Metrics

**Figure 6:**
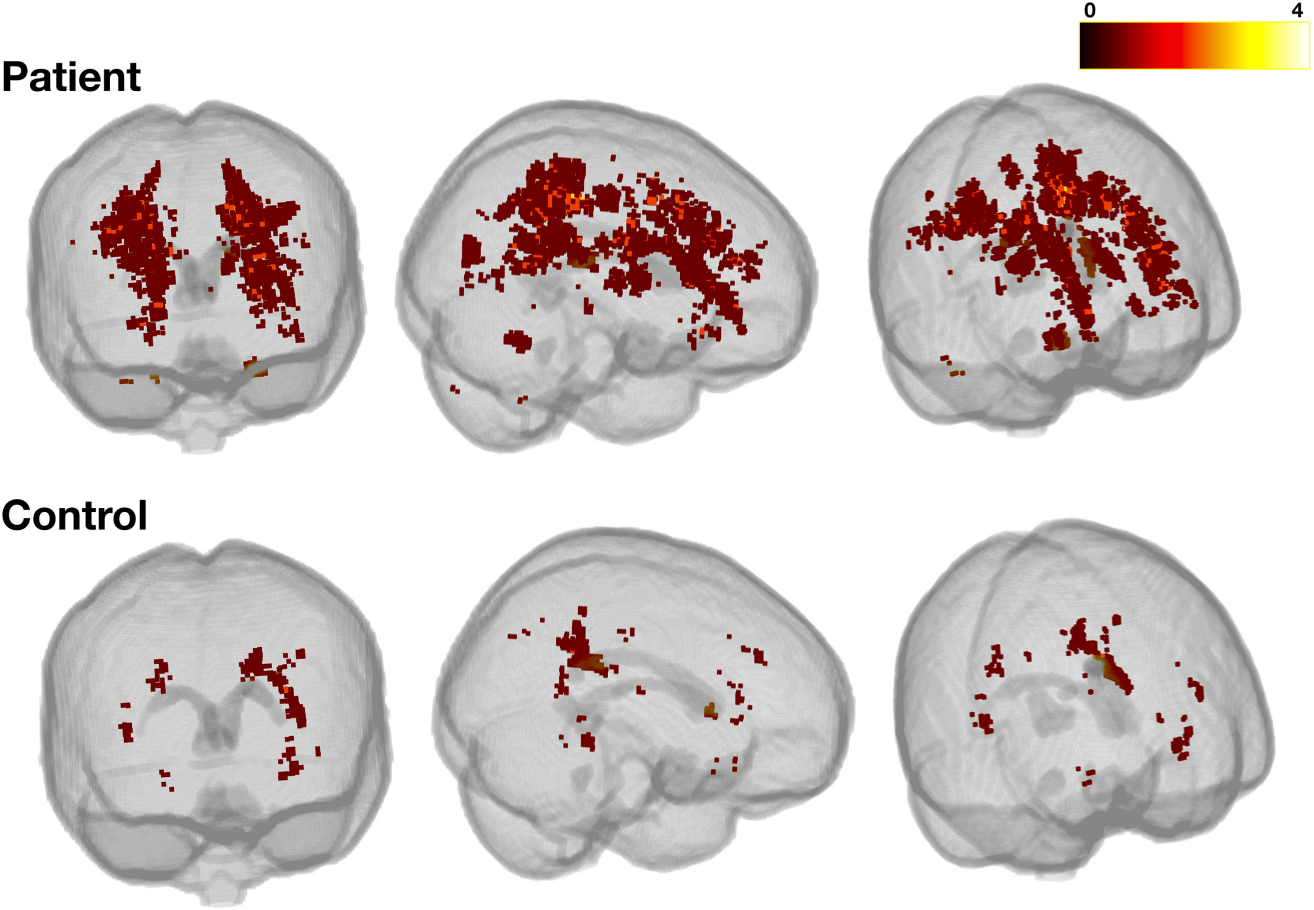
Heatmap for liberally-defined SCI. Showing the distribution and frequency of lesions for the liberal definition in patients (top row) and controls (bottom row). All lesions were registered to a standard space template (MNI 152) using non-linear registrations from the *FSL_anat* pipeline (https://fsl.fmrib.ox.ac.uk/fsl/fslwiki/fsl_anat). The color bar shows the number of participants with lesions in a particular voxel.

**Figure 7:**
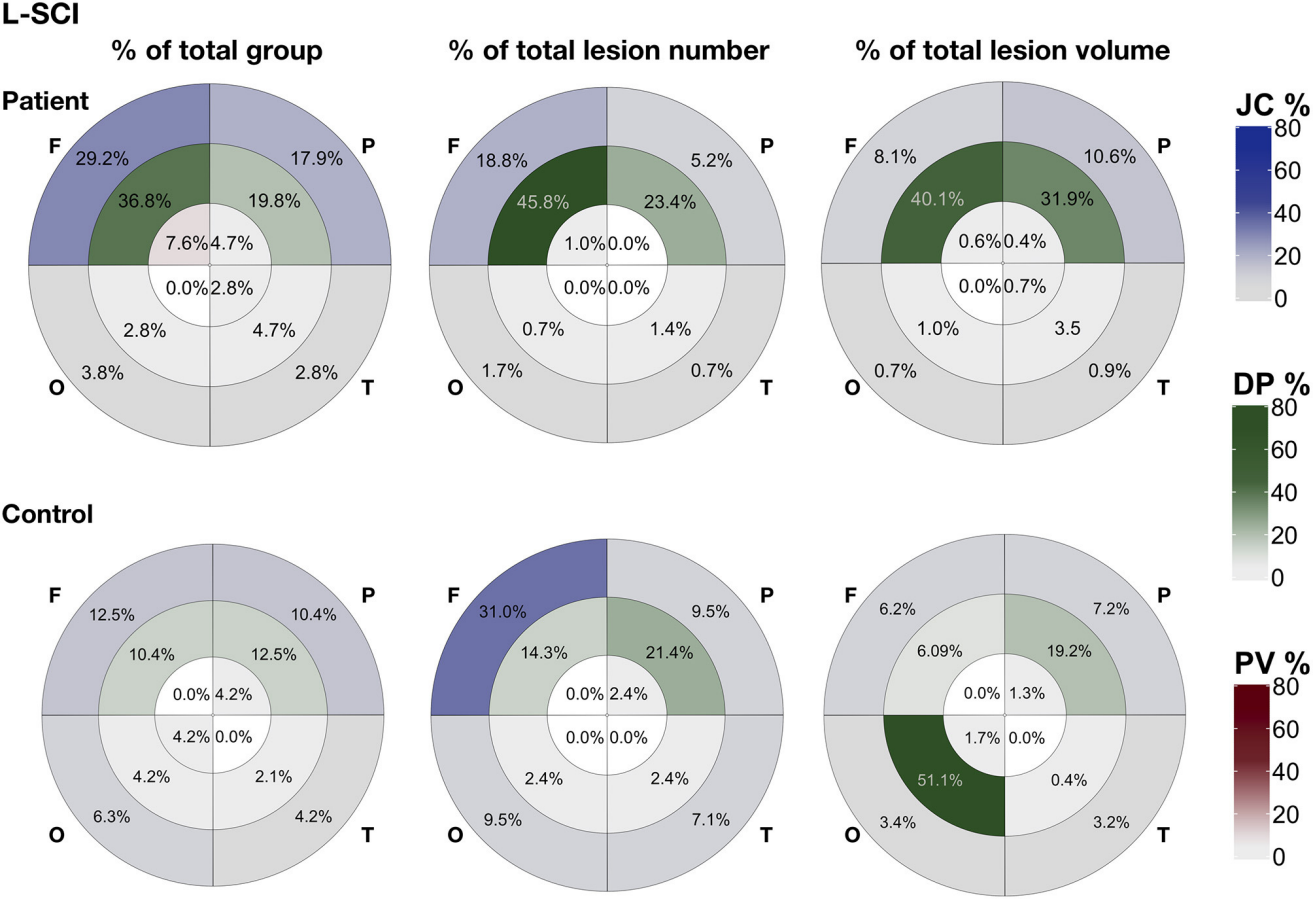
Lesion distribution for liberally-defined SCI. Showing the distribution of lesions using bullseye plots, where sectors represent the four lobes (F, frontal; P, parietal; O, occipital; T, temporal) and rings represent the three regions; periventricular (PV; interior ring; red), deep (DP; middle ring; green), and juxta-cortical (JC; exterior ring; blue), yielding 12 regional lobar zones. Distribution is represented using three different metrics for patients (top row) and controls (bottom row): (1) the proportion of the total group with lesion voxels in particular zone (% of total group), (2) the proportion of the total group lesion number classed as belonging to regional-lobar zone (middle - % of total lesion number), and (3) the proportion of the total group lesion voxels present in a particular regional-lobar zone (right - % of total lesion volume).

In patients, across SCI definitions and lesion metrics (Figures 6, 7, Supplementary Figures 2, 3), the distribution of lesions was relatively consistent. Following the L-SCI definition (Figures 6, 7), the deep frontal and parietal regional-lobar zones were the most commonly affected (36.8 and 19.8%, respectively), and accounted for the greatest proportion of the total number (69%) and volume (72%) of lesions in people with SCA. The juxta-cortical frontal and parietal zones were also among the most commonly affected in people with SCA (29.2 and 17.9%, respectively), but accounted for a relatively smaller proportion of the total number (24%) and volume (19%) of lesions, with the remaining regional-lobar zones rarely affected (all < 10%). In controls, there was far less consistency between lesion metrics based on the L-SCI definition. Whilst the juxta-cortical frontal zone accounted for the greatest proportion of the total lesion number (31%; Figure 7), the deep occipital zone accounted for the greatest proportion of lesion volume (51%; Figure 7). SCI were not particularly common in either zone, with the juxta-cortical frontal zone affected in six controls (12.5%), and the deep occipital zone in two (4.2%; Figure 7).

Lesion distributions in both patients and controls were similar between the L-SCI (Figure 7) and T-SCA definitions (Supplementary Figure 2) but varied with the R-SCI definition (Supplementary Figure 3). Although the overall pattern was similar in patients, frontal lesions were more likely to survive the R-SCI criteria. There was no obvious pattern as to the location of the two surviving lesions in controls.

### Regional SCI Metrics and Cognition

Exploratory regression models estimating cognitive performance from regional and lobar lesion metrics yielded results similar to those using global lesion metrics (Supplementary Table 1), although a couple of significant effects emerged in models predicting PSI. First, in models using regional lesion metrics to predict PSI, there was a significant negative effect of the presence of juxta-cortical lesions for the R-SCI definition. The effect was significant across the whole sample, and in the patient subsample, but was modest in size (r = 0.16–0.19), and disappeared in the more complex models (i.e., models 2 and 3; Table 1), where a significant positive effect of restrictively-defined deep lesion volume emerged in the SCA subsample (Supplementary Table 1). Second, in complex models using lobar lesion metrics to predict PSI, there were significant negative effects of parietal lesion volume for both the L-SCI and T-SCI definitions in the SCA subsample, but not for the R-SCI definition, or for any definition across the whole sample. For the R-SCI definition, a positive effect of frontal lesion volume was, however, observed in the SCA subsample.

Across all models, coefficients for the lesion volume variables that reached significance were very small in magnitude, corresponding to a 0.17–0.19 decrease in PSI points for every 10% increase in L-SCI or T-SCI defined parietal lesion volume, and a 0.43–0.44 increase in PSI for every 10% increase in restrictively defined frontal or deep lesion volume (Supplementary Table 1). Effect sizes were also modest (r = 0.20–0.22).

## Discussion

Using high-resolution FLAIR, we assessed the effect of three published SCI definitions on global and regional lesion metrics and explored their value in predicting general cognitive performance. As hypothesized, we observed large effects of SCI definition on estimates of lesion metrics in both patients and controls. However, contrary to hypotheses, neither the presence, nor the total number, nor the total volume of lesions based on any SCI definition predicted general cognitive performance or impairment. These findings underscore the numerous challenges surrounding the widespread use of binary SCI status as a biomarker of cognition in cross-sectional high-resolution MRI studies in the SCA field, including challenges related to the definition, quantification, distribution, specificity, and timing. Taken together, our findings suggest that with high-resolution MRI: (1) radiological definitions have a large effect on resulting lesion groups, numbers, and volumes; (2) there is a non-negligible prevalence of lesions also in healthy controls; and (3) at the group-level, there is no association between global lesion metrics and general cognitive outcome irrespective of lesion definition and metric.

### SCI Definitions and Global Lesion Metrics

We observed large effects of SCI definition on global lesion metrics, with the resulting lesion subgroups varying widely, particularly in controls. Estimates of SCI prevalence based on the L-SCI and T-SCI definitions in patients were in broad agreement (43% and 40%, respectively), and were broadly comparable with prior SCA studies employing higher field-strength MRI (43). Prevalence in patients based on the R-SCI definition was significantly lower (24%) but in line with a prior lower-resolution pediatric study (18). The total number and volume of lesions were less consistent between definitions, with 69 patient lesions (24%) and 26 control lesions (62%) not meeting the T-SCI definition, and a further 158 patient lesions (55%) and 14 control lesions (33%) not meeting the R-SCI definition. Of note, excluded lesions were often from individuals who had other lesions meeting the T1w hypointensity and/or minimum length criteria.

While radiological SCI definitions that include minimum length criteria may be justifiable in studies employing lower-resolution sequences where reliability may be a concern, there is less justification in higher resolution studies unless increased sensitivity to a specific pathology and/or outcome can be demonstrated. For prediction of cognitive performance, the present study indicated little utility of the length requirement of the T-SCI definition, or the additional T1 hypo-intensity requirement of the R-SCI definition, though both definitions had significant effects on resulting lesion groups, numbers, and volumes. These findings demonstrate the need for a standardized definition and quantification approach that is reliable and replicable for further exploration in high-resolution MRI studies. Ideally, future studies should also take into account the definitions used in studies of cerebral small vessel disease (CSVD) in adults (44–46).

For comparability with studies in CSVD, and to avoid excluding smaller lesions before attempting to explore and understand the features of such lesions, it may be useful to consider the greatest dimension and T1-intensity not as criteria for inclusion, but as features worthy of further exploration alongside other imaging features. Future studies should therefore consider employing L-SCI definitions and semi-automated segmentation techniques. Although substantial progress has been made in the development of automated segmentation techniques in other populations (47), the sensitivity of these techniques to the often smaller and less well-defined lesions observed in younger populations with and without SCA has yet to be investigated.

### Global Lesion Metrics and Cognitive Performance

Globally, our results indicate greater variation in cognition within than between lesion groups, with no observable dose-dependent effect of the total lesion burden or the lesion burden for age, in patients or controls. These findings align with those of several recent high-resolution MRI studies that have failed to detect an effect of the presence of T-SCI on cognition in patients with SCA (15, 16, 48) (Table 5). However, they contradict lower-resolution pediatric MRI studies where small to moderate effects have been widely reported (Supplementary Table 2) (10, 11, 28, 55, 56). These lower resolution studies include children enrolled in the Co-operative Study of Sickle Cell Disease (CSSCA) from 1989 to 1999 (56), and a subset of those enrolled in the SIT trial from 2005 to 2013 (42). Given their relatively large samples, prior meta-analyses have been heavily weighted by their inclusion [e.g., (10)].

**Table 5:** Overview of high-resolution MRI studies examining impact of SCI on cognitive outcomes in patients with SCA.

**Study details**			**SCA (N)**	**SCA (age)**	**SCI definition**	**MRI**	**Sequence Res**.	**Lesion Metric**	**% SCA w/ SCI**	**Cognitive Tests**	**Neg. effect of SCI—FSIQ, WMI, or PSI**	**Neg. effect of SCI—any domain**	**Results: Assoc w/cognition or other neuroimaging outcome**
Hijmans et al. (14)	Netherlands	2010	21	6–18 y	*No explicit definition*	1.5T, 3T	*Not reported*	Prevalence (i.e., binary y/n)	57%	WISC-III (composite scores), Stop-task, Tower of London, N-back task, Beery VMI	N	N	No significant differences found between SCI+ and SCI– groups
Krejza et al. (49)	US	2012	46	3–13 y	*No explicit definition*	3T	*Not reported*	Prevalence (i.e., binary y/n)	34%	K-BIT (Kaufman Brief Intelligence Test)	–	N	No significant differences in any K-bit score between SCI+ and SCI- groups, lesion volume included in models as covariate; significance and effect sizes not reported
van der Land et al. (29)	Netherlands	2015	38	8–17.1 y	L-SCI: area of abnormally increased signal on T2 and FLAIR	3T	T2 = 0.58 × 0.72 mm, 29 slices with 5 mm thickness; FLAIR = 1.03 × 1.68 mm, 29 slices with 5 mm thickness.	Prevalence (i.e., binary y/n) and volume (rank score)	50%	WISC-III (composite scores), Trail-making test, Beery VMI	Y	Y	SCI+ group significantly reduced FSIQ (81 vs. 89), VIQ (84 vs. 93), and PSI (83 vs. 97). Effects of SCI volume rank also observed in regression models for these scores.
Chen et al. (50)	US	2017	25	Decline M = 9.67, SD = 1.41. No Decline M = 9.38, SD = 2.94	T-SCI: area of abnormally increased signal on FLAIR—min 3 mm greatest diameter		FLAIR = 1 × 1 × 1 mm	Prevalence (i.e., binary y/n)	56%	K-BIT	–	N	SCI presence did not improve model fits for IQ decline over time
Downes et al. (51)	Ireland	2020	28	8–18 y	*No explicit definition*	*Not reported*	*Not reported*	Prevalence (i.e., binary y/n)	36%	NIH toolbox tests of executive function, language, and memory	N	N	No significant differences between SCI+ and SCI- groups in EF or memory tasks, significantly better performance in the picture vocabulary task
Stotesbury et al. (16)	UK	2018	83	8–37 y	T-SCI: area of abnormally increased signal T2 and FLAIR—min 3 mm greatest diameter	3T	FLAIR = 0.65 × 1 × 0.65 mm, T2 = 0.51 × 0.51 × 5.6 mm	Prevalence (i.e., binary y/n)	45%	WASI, WAIS/WISC-IV (composite scores)	N	N	No significant differences found between SCI+ and SCI- groups in FSIQ or PSI
Hood et al. (52)^∧^	US	2019	61	3–22 y	T-SCI	3T	*Not reported*	Prevalence (i.e., binary y/n)	41%	NIH toolbox tests of executive function, language, and memory	N	N	No significant differences found between SCI+ and SCI- groups for overall cognition (83.12 vs. 83.92), executive function (90.81 vs. 87.90) or non-executive function (89.51 vs. 91.90) composites
Choi et al. (15)	US	2019	52	M = 21.4, SD = 7.7	T-SCI w/age: increased signal FLAIR—min 3 mm—>1 per decade of age	3T	FLAIR = 1.3 × 1.0 × 1.0 mm	Prevalence (i.e., binary y/n)	48%	WASI, WISC-IV	N	N	No significant differences between those with a normal and abnormal burden of SCI for age observed for FSIQ or matrix reasoning (others not reported)
Farris et al. (53)	US	2015	15	18–55 y	L-SCI: area of abnormally increased signal on FLAIR	3T	*Not reported*,	Number	60%	Cogstate battery: GMLT	–	N	No association between SCI number and performance on GMLT
Farris et al. (48)	US	2016	15	18–55 y	L-SCI: area of abnormally increased signal on FLAIR	3T	*Not reported, but slice thickness mentioned as included in volume estimate*	Volume	–	Cogstate battery: GMLT	–	N	No association between SCI volume and performance on GMLT
Sanger et al. (54)	US	2016	48	19–59 y	*No explicit definition*	–	*Not reported*	Prevalence (i.e., binary y/n)	37.5%	–	–	–	No differences in SCI prevalence as a function of employment status

*Studies identifies from Pubmed searches in July 2021 using the terms “sickle” paired with “cognitive,” or “IQ” or “executive function” or “MRI”—supplemented with literature known to the co-authors. Note: there may be between-study overlap in participants. ∧personal communication with author. SCA, Sickle Cell Anemia; C, Control; Res, Resolution; N, no; Y, yes; WISC/WAIS, Wechsler intelligence scale for children/ adults; WASI, Wechsler abbreviated scale of intelligence; K-BIT, Kaufman Brief Intelligence Test; GMLT, Groton Maze Learning Test of executive function; VMI, visual motor integration; L-/T-/R-SCI, Liberal/Traditional/Restrictive silent cerebral infarction definitions*.

Of 11 studies conducted at 3T examining effects of SCI on cognitive outcome, the only study to detect an effect used a sequence with a 5 mm slice thickness (Table 5), while 16 of 26 studies conducted at 1.5T found a negative effect of SCI on cognition (Supplementary Table 2). Taken together, these findings are consistent with the notion that the use of higher magnetic field strengths (i.e., 3T vs 1.5T) and higher resolution FLAIR sequences (i.e., 0.65 × 0.65 × 1 mm vs. 1.8 × 1.8 × 5 mm) increases lesion detectability, and that this in turn may skew or nullify prediction of cognitive impairment based on a binary categorization according to presence or absence of SCI, irrespective of the stringency of the definition employed. Increased lesion detectability is consistent with the relatively large proportion of controls we identified with SCI meeting L-SCI and T-SCI criteria (23 and 10%, respectively), the majority of whom were children. Other recent high-resolution MRI studies have similarly reported a non-negligible prevalence of T-SCI in healthy young adults (Table 6).

**Table 6:** Overview of SCI prevalence in prior high-resolution imaging studies in patients with SCA and controls.

**Study details**			**SCA (N)**	**SCA (age)**	**C (N)**	**C (age)**	**SCI definition**	**MRI**	**Sequence Res**.	**% SCA w/SCI**	**%C w/SCI**
Farris et al. (53)	US	2015	15	18–55 y	7	18–55 y	L-SCI: area of abnormally increased signal on FLAIR	3T	*Not reported*,	60%	57%
van der Land et al. (22)	Netherlands	2015	10	18–25 y	10	19–25 y	L-SCI: area of abnormally increased signal on FLAIR and T2	7T	FLAIR = 0.8 mm isotropic T2 = 0.7 mm isotropic	90%	70%
Choi et al. (26)	US	2017	33	11–41 y	32	12–41 y	T-SCI w/age: area of abnormally increased signal FLAIR - min 3mm - >1 per decade of age	3T	FLAIR = 1.3 × 1.0 × 1.0 mm	39%	13%
Coloigner et al. (57)	US	2017	20	12–34 y	19	17–41 y	T-SCI w/age: area of abnormally increased signal FLAIR - min 3mm greatest diameter - more than 1 per decade of age	3T	FLAIR = 1.3 × 1.0 × 1.0 mm	20%	0%
Choi et al. (15)	US	2019	52	M = 21.4, SD = 7.7	40	M = 27.7, SD = 11.3	T-SCI w/age: increased signal FLAIR—min 3 mm – > 1 per decade of age	3T	FLAIR = 1.3 × 1.0 × 1.0 mm	48%	26%
Václavu et al. (58)	Netherlands + US	2020	36	M = 37.4, SD = 15.43	9	M = 32.08, SD = 11.14	T-SCI: area of abnormally increased signal on FLAIR—min 2 mm greatest diameter	3T	FLAIR = 0.98 × 0.98 × 1.12 mm	82%	45%
Chai et al. (59)	US	2021	26	M = 24.2, SD = 9.7	21	M = 22.6, SD = 8.9	T-SCI: area of abnormally increased signal on FLAIR—min 3 mm greatest diameter	3T	FLAIR = 1.3 × 1 × 1 mm	54%	33%
Wang et al. (60)	US	2021	34	19–28 y	49	28–37 y	*No explicit definition*	3T	FLAIR = 1.0 × 0.9 × 3.0 mm	76%	39%

*Studies identifies from Pubmed searches in July 2021 using the terms “sickle” paired with “cognitive,” or “IQ” or “executive function” or “MRI”—supplemented with literature known to the co-authors. There may be between-study overlap in participants. SCA, Sickle Cell Anemia; C, Control; Res, Resolution; L-/T-/R-SCI, Liberal/Traditional/Restrictive silent cerebral infarction definitions*.

This interpretation raises the possibility that there may be a tipping point beyond which lesions become large and prominent and begin to have an effect. It is also possible that the inclusion of young adults in our sample reduced our sensitivity to potential age-dependent effects of SCI across specific domains. Although we excluded participants >30 years, used cognitive scores that were scaled for age, included age as a covariate in our regression models, and observed no relationships between age and global lesion metrics, we cannot exclude the possibility of age-dependent effects of the global presence and burden of lesions on individual cognitive trajectories in specific domains.

Discrepancies in the literature may also be accounted for by differences in sample characteristics between prior and more recent studies. The lesions observed at 1.5T MRI in the majority of prior studies are likely to have met the traditional or restrictive criteria (Supplementary Table 2). These studies included untreated patients with clinically significant cerebral vasculopathy, which may have contributed to both their SCIs and their cognitive deficits. Therapies such as hydroxyurea and transfusion were rarely used during the CSSCA but were prescribed in 40 and 21%, respectively of our cohort with SCA. In a recent study, executive function improved soon-after compared with long-after a blood transfusion in 27 people with SCA, >80% of whom had infarction on MRI (52). However, although the incidence of recurrent infarcts, including overt strokes and T-SCIs, was reduced, blood transfusion for 3 years did not improve IQ in SIT (3). One of the exclusion criteria was treatment with hydroxyurea within 3 months of SIT enrollment but there is now evidence for a protective effect on IQ (61, 62). There is therefore some evidence that the discrepancy between our data and previous studies could be related to therapy. It is also possible that the strategic position of SCI in relation to particularly eloquent tracts plays a role in functional outcomes.

### Regional Lesion Metrics and Cognitive Performance

Our regional analyses yielded several interesting findings. We detected a consistent regional distribution of lesions across metrics and definitions in patients, but not controls. Whilst lesions in patients were similarly prevalent in deep and juxta-cortical frontal and parietal zones, lesions in deep frontal and parietal zones were greater in number and size, echoing prior SCA studies (4, 63, 64). In exploratory models, we detected some significant effects of regional and lobar lesion metrics on PSI. The most consistent finding was a moderate negative effect on PSI of parietal lesion volume for both the L-SCI and T-SCI definitions in the SCA subsample. However, confidence intervals were wide, and coefficients very small for these effects, highlighting the need to interpret them with caution. Findings for the restrictive definition were less consistent. There was a moderate negative effect of the presence of juxta-cortical R-SCI in simple models, but the effect disappeared in the more complex models, where moderate positive effects of deep and frontal R-SCI volume emerged, again with wide confidence intervals and small coefficients.

It is possible that the paradoxical effects we observed using the R-SCI definition were related to our operationalization of the visual definition of a T1-hypointensity (18), which was novel and may have been too stringent. Given that the R-SCI definition requires a corresponding FLAIR hyperintensity, and we applied the T1 threshold after the FLAIR threshold, it is also possible that some T1 hypo-intensities were missed and/or excluded (Figure 1). Alternatively, T1 hypo-intensities may represent older, more established lesions, with effects diminishing over time.

These possibilities could also account for the paradoxical univariate association observed between higher volume and larger number of R-SCI and higher, rather than lower, IQ in children with SCA <16 years. Another possibility is that in the child's brain, adaptive physiology which may enable brain growth and development, e.g., higher cerebral blood flow (CBF), might eventually favor the development of SCI in the borderzone regions (64, 65) related to vascular instability secondary to exhaustion of cerebral reserve mechanisms, shunting or steal. Such effects may plausibly improve or worsen over time depending on age, along with the severity of the disease course. It might be possible to follow the trajectory in patients longitudinally from a young age by comparing the prevalence, number and distribution of T-SCI, L-SCI, and R-SCI. This would enable exploration of trajectories and could help establish whether R-SCI, defined by T1 hypointensities, represent older, more established lesions.

### Limitations

It is worth re-iterating that for the few effects of SCI that we did detect, there was considerable variability with associated small coefficients and modest *p*-values. Given the high number of models computed, these effects would not have survived correction for multiple comparisons, irrespective of method. In line with prior studies [e.g., (18)], and as we wanted to maximize our ability to detect effects of SCI should they be present, we elected not to apply correction for multiple comparisons in this study. For this reason, it is particularly noteworthy that despite not correcting for multiple comparisons, we observed no effects of SCI metrics on cognitive difficulties in our global models. One drawback to this approach is that the few effects that we did detect in univariate correlations and regional models may have been spurious.

Beyond our decision to not correct for multiple comparisons, limitations of our study include our lack of direct measures of socio-economic status and of hemoglobin in controls. Unless direct benefit can be demonstrated, invasive measurements are often considered inappropriate by UK ethical committees. Our patient sample was also heterogeneous, with patients on a mix of disease-modifying treatment types (i.e., hydroxyurea and chronic transfusion regimens) for unknown and likely variable durations. These limitations may have decreased the sensitivity of our regression models, but given the raw data, are unlikely to have had a major impact on the results. Unlike prior studies (2), we also did not have the resources for central adjudication of SCIs across a panel of neuroradiologists. This limitation was partially mitigated by our neuroradiologist's expertise, along with our use of a semi-automated pipeline, which ensured that previously-established (29) intensity thresholds were consistently applied.

A further limitation is that we did not include further multi-modal analyses on the etiology or consequences of SCI in this manuscript. It is however worth noting that although the name “SCI” implies infarction, white matter hyperintensities on FLAIR are non-specific, and other underlying pathologies are possible, both in patients and controls. Although the pathophysiology is unknown, the distinction between periventricular, deep, and juxta-cortical lesions is thought to be etiologically meaningful (66). The anatomical distinction is however based on relatively arbitrary distance thresholds, and there is scope for further optimization. Exploring regional and lobar effects is challenging in SCA because most patients have multiple lesions, often affecting multiple regional-lobar zones, and there is substantial variability in terms of lesion number, volume, and spatial distribution. Given that our findings suggest a peak voxel lesion density of 9% (*n* = 4; Figure 5), the statistical power for voxel-wise approaches is likely to be limited.

### Future Directions

Using quantitative multi-modal methods to explore the haemodynamic, susceptibility, and microstructural characteristics of lesions, and of normal-appearing white-matter in anatomical regions “at-risk” of haemodynamic compromise (63, 65, 67–69), may enable better lesion classification, and may also shed further light on the underlying pathology. Understanding relationships between the microstructural tissue characteristics of lesions and normal-appearing white matter is particularly relevant in SCA, where studies have shown widespread and functionally significant reductions in the integrity of normal-appearing white matter (16, 70–72), also in patients without lesions. Another advantage of quantitative multi-modal approaches is that they provide continuous measures that are more normally distributed, and more powerful to work with statistically.

In parallel with quantification of SCI, documenting the effect of reduced arterial oxygen content secondary to anemia and hypoxia on cerebral blood flow (65) and oxygen extraction fraction (73), may shed light on the etiology of cognitive difficulties in SCA, alongside connectivity studies (74). In addition, regional brain volumes may be reduced in SCA (75, 76); we did not find an effect of total intracranial volume in our regressions but further regional analyses are planned.

Future quantitative multi-modal approaches may also help overcome another major challenge, which is that lesions are not detected at the time they occur. As a result, there is no way to determine the presence or absence of transient focal neurological symptoms at the time of insult. Elegant work has demonstrated a high prevalence of lesions on diffusion-weighted imaging in acutely anemic, as well as in steady-state (77) patients with SCA, with only some of these acute lesions later transitioning into SCI observable on FLAIR. Longitudinal quantitative multi-modal MRI studies may help identify what determines this tipping point. Such studies may also shed light on whether acute lesions are reversible or produce permanent damage below the resolution of qualitative MRI.

## Conclusion

In conclusion, our results highlight the challenges surrounding SCI definition and quantification and suggest limited utility of SCI metrics as biomarkers of cognitive dysfunction in cross-sectional high-resolution MRI studies in patients with SCA. We have demonstrated that with high-resolution MRI: (1) radiological definitions have a large effect on resulting lesion groups, numbers, and volumes; (2) there is a non-negligible prevalence of lesions in young healthy race-matched controls; and (3) at the group-level, there is no cross-sectional association between global lesion metrics and general cognitive outcome irrespective of lesion definition and metric. As high-resolution multi-modal MRI is more widely available, the dichotomy of presence or absence of T-SCI does not appear to be a sensitive biomarker for detection of functionally significant pathology. There is therefore a need to move beyond the focus on the SCI dichotomy, with further standardization regarding definitions and methods, toward a better understanding of the broader underlying pathology. As such, the search should continue for appropriate endpoints for clinical treatment trials, including brief cognitive assessments in domains important for school and work performance, such as processing speed and executive function (52) as well as patient-reported outcomes (e.g., fatigue, pain).

## Data Availability Statement

The data supporting the conclusions of this article will be made available by the authors, without undue reservation.

## Ethics Statement

The studies involving human participants were reviewed and approved by West London NHS (SAC; 05/Q0408/42, 11/EM/0084, 15/LO/0347), Yorkshire NHS, (POMS; 15/YH/0213), and University College London (community recruitment 14475/001) research Ethics Committees. Written informed consent to participate in this study was provided by participants or their legal guardian/next of kin.

## Author Contributions

HS: conceptualization, data curation, investigation, methodology, formal analysis, visualization, and writing—original draft. JK: conceptualization, investigation, methodology, and writing—review and editing. JC: resources, methodology, formal analysis, validation, and writing—review and editing. DS: investigation, and writing—review and editing. AH: resources, methodology, and writing—review and editing. MK: data curation, investigation, and writing—review and editing. SS: data curation, investigation, and project administration. DR, OW, ML, MP, BI, JH, and SC: investigation and writing—review and editing. CC: supervision and writing—review and editing. FK: conceptualization, investigation, supervision, funding acquisition, methodology, and writing—review and editing. All authors contributed to the article and approved the submitted version.

## Funding

HS and MK were funded by Action Medical Research (GN2509) and JK by Great Ormond Street Children's Charity (V4615). AH was supported by an NIH grant (1F32HL143915). The National Institute for Health Research (PB-PG-1112-29099) and NIH (R01HL079937) provided funding for patient recruitment. The work was supported by the National Institute for Health Research Biomedical Research Centre (IS-BRC-1215-20012) at Great Ormond Street Hospital for Children NHS Foundation Trust and University College London.

## Conflict of Interest

FK was grantholder for GN2509, V4615, PB-PG-1112-29099 and R01HL079937 and has received honoraria from Global Blood Therapeutics, Bluebird Bio, Novartis, BIAL, Shire and Johnson and Johnson. JH received research funding from Bluebird Bio, and payments in relation to work as an advisory board member from IMR, Novartis, Global Blood Therapeutics, Novo Nordisk, Forma therapeutics, Agios, Add Medica, and Terumo, and also received a travel grant from Novartis and payments relating to work as a panel speaker from Novartis and Global Blood Therapeutics. The remaining authors declare that the research was conducted in the absence of any commercial or financial relationships that could be construed as a potential conflict of interest.

## Publisher's Note

All claims expressed in this article are solely those of the authors and do not necessarily represent those of their affiliated organizations, or those of the publisher, the editors and the reviewers. Any product that may be evaluated in this article, or claim that may be made by its manufacturer, is not guaranteed or endorsed by the publisher.

## Appendix

**Table A1:** Global regression models.

**Global Models**	**1) SCI**	**2) SCI*** **SCA**		**3) SCI** **+** **SCI No**.		**4) SCI** ***** **SCA** **+** **SCI No** ***** **SCA**				**5) SCI** **+** **SCI Vol**		**6) SCI** ***SCA** **+** **SCI Vol** ***** **SCA**			
***Predictors***	**SCI**	**SCI**	**SCI* SCA**	**SCI**	**SCI No**.	**SCI**	**SCI* SCA**	**SCI No**.	**SCI No. * SCA**	**SCI**	**SCI Vol**	**SCI**	**SCI *SCA**	**SCI Vol**	**SCI Vol * SCA**
**FSIQ**															
***L-SCI*** *any SCI* *(all, n = 154)*	b = −0.59, *p =* 0.79, r = −0.02, CI = −5.0–3.8	b = −0.29, *p =* 0.90, r = −0.01, CI = −4.8–4.2	b = −0.54, *p =* 0.92, r = −0.01, CI = −10.7–9.7	b = 0.73, *p =* 0.82, r = 0.02, CI = −5.8–7.2	b = −1.04, *p =* 0.58, r = −0.04, CI = −4.8–2.7	b = 2.21, *p =* 0.52, r = 0.05, CI = −4.6–9.0	b = −7.85, *p =* 0.34, r = −0.08, CI = −24.0–8.3	b = −2.32, *p =* 0.29, r = −0.09, CI = −6.6–2.0	b = 6.83, *p =* 0.23, r = 0.10, CI = −4.3–18.0	b = 1.71, *p =* 0.69, r = 0.03, CI = −6.8–10.2	b = −0.59, *p =* 0.53, r = −0.05, CI = −2.4–1.3	b = 2.08, *p =* 0.64, r = 0.04, CI = −6.6–10.7	b = −5.15, *p =* 0.58, r = −0.04, CI = −23.7–13.4	b = −0.74, *p =* 0.47, r = −0.06, CI = −2.7–1.3	b = 1.61, *p =* 0.49, r = 0.05, CI = −3.0–6.3
*>1 SCI per decade (all, n = 154)*	b = 0.06, *p =* 0.98, r = 0.00, CI = −4.6–4.7	b = 0.09, *p =* 0.97, r = 0.00, CI = −4.7–4.8	b = 1.98, *p =* 0.72, r = 0.03, CI = −9.0–13.0	–	–	–	–	–	–	–	–	–	–	–	–
*Any SCI* *(SCA only, n = 106)*	b = −0.04, *p =* 0.99, r = −0.00, CI = −5.3–5.2	–	–	b = 1.01, *p =* 0.79, r = 0.02, CI = −6.6–8.7	b = −0.77, *p =* 0.71, r = −0.04, CI = −4.9–3.3	–	–	–	–	b = 2.50, *p =* 0.64, r = 0.04, CI = −8.0–13.0	b = −0.60, *p =* 0.58, r = −0.05, CI = −2.8–1.6	–	–	–	–
***T-SCI*** *any SCI* *(all, n = 154)*	b = −0.07, *p =* 0.98, r = −0.00, CI = −4.8–4.6	b = −0.12, *p =* 0.96, r = −0.00, CI = −5.4–5.1	b = 3.53, *p =* 0.60, r = 0.04, CI = −9.6–16.6	b = 1.03, *p =* 0.76, r = 0.02, CI = −5.7–7.8	b = −0.96, *p =* 0.65, r = −0.04, CI = −5.2–3.3	b = 1.37, *p =* 0.73, r = 0.03, CI = −6.6–9.3	b = 2.58, *p =* 0.81, r = 0.02, CI = −18.7–23.8	b = −1.43, *p =* 0.64, r = −0.04, CI = −7.5–4.6	b = 1.34, *p =* 0.88, r = 0.01, CI = −16.2–18.9	–	b = −0.21, *p =* 0.67, r = −0.03, CI = −1.2–0.8	–	–	b = −0.25, *p =* 0.67, r = −0.03, CI = −1.4–0.9	b = 0.64, *p =* 0.68, r = 0.03, CI = −2.4–3.7
*>1 SCI per decade (all, n = 154)*	b = 0.34, *p =* 0.89, r = 0.01, CI = −4.7–5.3	b = 0.11, *p =* 0.97, r = 0.00, CI = −5.3–5.5	b = 3.84, *p =* 0.57, r = 0.05, CI = −9.4–17.0	–	–	–	–	–	–	–	–	–	–	–	–
*Any SCI* *(SCA only, n = 106)*	b = 1.27, *p =* 0.64, r = 0.04, CI = −4.0–6.6	–	–	b = 3.30, *p =* 0.39, r = 0.08, CI = −4.3–10.9	b = −1.68, *p =* 0.45, r = −0.07, CI = −6.1–2.8	–	–	–	–	–	b = −0.08, *p =* 0.89, r = −0.01, CI = −1.2–1.0	–	–	–	–
***R-SCI*** *any SCI* *(all, n = 154)*	b = 0.28, *p =* 0.92, r = 0.01, CI = −5.3–5.9	–	–	b = −0.44, *p =* 0.90, r = −0.01, CI = −7.5–6.6	b = 1.29, *p =* 0.74, r = 0.03, CI = −6.3–8.9	–	–	–	–	b = −1.91, *p =* 0.65, r = −0.04, CI = −10.1–6.3	b = 1.21, *p =* 0.47, r = 0.06, CI = −2.1–4.5	–	–	–	–
*>1 SCI per decade (all, n = 154)*	b = 3.35, *p =* 0.41, r = 0.07, CI = −4.59–11.29	–	–			–	–	–	–	–	–	–	–	–	–
*Any SCI* *(SCA only, n = 106)*	b = 0.58, *p =* 0.85, r = 0.02, CI = −5.5–6.6	–	–	b = 0.48, *p =* 0.90, r = 0.01, CI = −7.4–8.3	b = 0.17, *p =* 0.97, r = 0.00, CI = −7.8–8.2	–	–	–	–	b = −1.41, *p =* 0.75, r = −0.03, CI = −10.3–7.5	b = 1.04, *p =* 0.55, r = 0.06, CI = −2.4–4.5	–	–	–	–
**WMI** ***L-SCI*** *any SCI* *(all, n = 154)*	b = −0.22, *p =* 0.93, r = −0.01, CI = −5.1–4.61	b = 0.11, *p =* 0.97, r = 0.00, CI = −4.81–5.03	b = 3.14, *p =* 0.58, r = 0.04, CI = −7.94–14.21	b = 2.16, *p =* 0.55, r = 0.05, CI = −4.95–9.26	b = −1.86, *p =* 0.37, r = −0.07, CI = −5.93–2.21	b = 2.63, *p =* 0.49, r = 0.06, CI = −4.81–10.06	b = 4.27, *p =* 0.63, r = 0.04, CI = −13.3–21.81	b = −1.97, *p =* 0.40, r = −0.07, CI = −6.64–2.69	b = −0.50, *p =* 0.94, r = −0.01, CI = −12.6–11.63	b = 3.39, *p =* 0.47, r = 0.06, CI = −5.91–12.68	b = −0.92, *p =* 0.37, r = −0.07, CI = −2.95–1.11	b = 3.78, *p =* 0.43, r = 0.06, CI = −5.60–13.15	b = 9.34, *p =* 0.36, r = 0.07, CI = −10.74–29.42	b = −0.85, *p =* 0.44, r = −0.06, CI = −3.01–1.30	b = −1.51, *p =* 0.55, r = −0.05, CI = −6.54–3.52
*>1 SCI per decade (all, n = 154)*	b = −0.83, *p =* 0.75, r = −0.03, CI = −5.94–4.27	b = −0.64, *p =* 0.81, r = −0.02, CI = −5.81–4.52	b = 1.83, *p =* 0.76, r = 0.02, CI = 10.14–13.79	–	–	–	–	–	–	–	–	–	–	–	–
*Any SCI* *(SCA only, n = 106)*	b = 1.56, *p =* 0.59, r = 0.05, CI = −4.10–7.21	–	–	b = 5.14, *p =* 0.22, r = 0.12, CI = −3.11–13.39	b = −2.63, *p =* 0.24, r = −0.11, CI = −7.05–1.79	–	–	–	–	b = 8.37, *p =* 0.15, r = 0.14, CI = −3.0–19.7	b = −1.61, *p =* 0.17, r = −0.13, CI = −4.0–0.7	–	–	–	–
***T-SCI*** *any SCI* *(all, n = 154)*	b = 0.46, *p =* 0.86, r = 0.01, CI = −4.68–5.59	b = 1.22, *p =* 0.67, r = 0.03, CI = −4.48–6.93	b = 1.56, *p =* 0.83, r = 0.02, CI = −12.68–15.79	b = 2.45, *p =* 0.51, r = 0.05, CI = −4.92–9.81	b = −1.74, *p =* 0.46, r = −0.06, CI = −6.34–2.87	b = 2.50, *p =* 0.57, r = 0.05, CI = −6.15–11.15	b = 5.52, *p =* 0.64, r = 0.04, CI = −17.5–28.58	b = −0.96, *p =* 0.77, r = −0.02, CI = −7.54–5.62	b = −3.60, *p =* 0.71, r = −0.03, CI = −22.6–15.40	–	b = −0.20, *p =* 0.72, r = −0.03, CI = −1.28–0.89	–	–	b = 0.01, *p =* 0.99, r = 0.00, CI = −1.26–1.28	b = −0.18, *p =* 0.91, r = −0.01, CI = −3.46–3.10
*>1 SCI per decade (all, n = 154)*	b = 0.09, *p =* 0.97, r = 0.00, CI = −5.37–5.55	b = 0.60, *p =* 0.84, r = 0.02, CI = −5.27–6.48	b = 0.70, *p =* 0.92, r = 0.01, CI = −13.66–15.06	–	–	–	–	–	–	–	–	–	–	–	–
*Any SCI* *(SCA only, n = 106)*	b = 2.08, *p =* 0.48, r = 0.07, CI = −3.67–7.83	–	–	b = 5.44, *p =* 0.19, r = 0.13, CI = −2.73–13.62	b = −2.79, *p =* 0.25, r = −0.11, CI = −7.60–2.03	–	–	–	–	–	b = −0.05, *p =* 0.93, r = −0.01, CI = −1.23–1.13	–	–	–	–
***R-SCI*** *any SCI* *(all, n = 154)*	b = 0.29, *p =* 0.93, r = 0.01, CI = −5.84–6.41	–	–	b = 1.82, *p =* 0.64, r = 0.04, CI = −5.86–9.51	b = −2.76, *p =* 0.51, r = −0.05, CI = −11.06–5.55	–	–	–	–	b = −1.45, *p =* 0.75, r = −0.03, CI = −10.41–7.51	b = 0.96, *p =* 0.60, r = 0.04, CI = −2.65–4.57	–	–	–	–
*>1 SCI per decade (all, n = 154)*	b = −1.30, *p =* 0.77, r = −0.02, CI = −10.01–7.41	–	–	–	–	–	–	–	–	–	–	–	–	–	–
*Any SCI* *(SCA only, n = 106)*	b = 1.13, *p =* 0.74, r = 0.03, CI = −5.46–7.72	–	–	b = 3.76, *p =* 0.38, r = 0.08, CI = −4.74–12.25	b = −4.25, *p =* 0.33, r = −0.09, CI = −12.91–4.41	–	–	–	–	b = −0.53, *p =* 0.91, r = −0.01, CI = −10.25–9.18	b = 0.87, *p =* 0.64, r = 0.04, CI = −2.86–4.60	–	–	–	–
**PSI**															
***L-SCI ________*** *Any SCI* *(all, n = 154)*	b = −1.67, *p =* 0.46, r = −0.06, CI = −6.09–2.75	b = −1.13, *p =* 0.62, r = −0.04, CI = −5.58–3.32	b = 2.30, *p =* 0.65, r = 0.03, CI = −7.72–12.32	b = −1.26, *p =* 0.70, r = −0.03, CI = −7.77–5.24	b = −0.32, *p =* 0.87, r = −0.01, CI = −4.05–3.41	b = 0.04, *p =* 0.99, r = 0.00, CI = −6.70–6.77	b = −1.14, *p =* 0.89, r = −0.01, CI = −17.0–14.75	b = −1.09, *p =* 0.61, r = −0.04, CI = −5.32–3.14	b = 3.21, *p =* 0.56, r = 0.04, CI = −7.78–14.21	b = 0.30, *p =* 0.94, r = 0.01, CI = −8.20–8.80	b = −0.50, *p =* 0.59, r = −0.04, CI = −2.36–1.35	b = 1.05, *p =* 0.81, r = 0.02, CI = −7.45–9.54	b = −0.99, *p =* 0.91, r = −0.01, CI = −19.2–17.22	b = −0.66, *p =* 0.51, r = −0.05, CI = −2.61–1.30	b = 1.19, *p =* 0.61, r = 0.04, CI = −3.37–5.76
*>1 SCI per decade (all, n = 154)*	b = −0.93, *p =* 0.69, r = −0.03, CI = −5.60–3.74	b = −0.83, *p =* 0.73, r = −0.03, CI = −5.50–3.83	b = 4.73, *p =* 0.39, r = 0.07, CI = −6.07–15.53	–	–	–	–	–	–	–	–	–	–	–	–
*Any SCI* *(SCA only, n = 106)*	b = −0.08, *p =* 0.97, r = −0.00, CI = −5.08–4.91	–	–	b = 0.52, *p =* 0.89, r = 0.01, CI = −6.82–7.86	b = −0.44, *p =* 0.82, r = −0.02, CI = −4.37–3.49	–	–	–	–	b = 2.20, *p =* 0.67, r = 0.04, CI = −7.92–12.32	b = −0.54, *p =* 0.61, r = −0.05, CI = −2.62–1.54	–	–	–	–
***T-SCI*** *any SCI* *(all, n = 154)*	b = −0.94, *p =* 0.69, r = −0.03, CI = −5.63–3.75	b = −0.35, *p =* 0.89, r = −0.01, CI = −5.51–4.81	b = 4.40, *p =* 0.50, r = 0.05, CI = −8.47–17.27	b = 0.32, *p =* 0.93, r = 0.01, CI = −6.42–7.06	b = −1.10, *p =* 0.61, r = −0.04, CI = −5.31–3.11	b = 0.59, *p =* 0.88, r = 0.01, CI = −7.24–8.41	b = 7.05, *p =* 0.51, r = 0.05, CI = −13.8–27.92	b = −0.72, *p =* 0.81, r = −0.02, CI = −6.68–5.24	b = −2.38, *p =* 0.78, r = −0.02, CI = −19.6–14.82	–	b = −0.38, *p =* 0.45, r = −0.06, CI = −1.37–0.61	–	–	b = −0.25, *p =* 0.67, r = −0.03, CI = −1.40–0.90	b = 0.50, *p =* 0.74, r = 0.03, CI = −2.46–3.47
*>1 SCI per decade (all, n = 154)*	b = −0.99, *p =* 0.69, r = −0.03, CI = −5.99–4.00	b = −0.75, *p =* 0.78, r = −0.02, CI = −6.05–4.56	b = 3.85, *p =* 0.56, r = 0.04, CI = −9.12–16.83	–	–	–	–	–	–	–	–	–	–	–	–
*Any SCI* *(SCA only, n = 106)*	b = 1.22, *p =* 0.63, r = 0.04, CI = −3.86–6.30	–	–	b = 3.40, *p =* 0.35, r = 0.09, CI = −3.84–10.64	b = −1.81, *p =* 0.40, r = −0.08, CI = −6.07–2.46	–	–	–	–	–	b = −0.11, *p =* 0.84, r = −0.02, CI = −1.15–0.94	–	–	–	–
***R-SCI*** *any SCI* *(all, n = 154)*	b = −2.56, *p =* 0.37, r = −0.07, CI = −8.15–3.03	–	–	b = −1.91, *p =* 0.59, r = −0.04, CI = −8.93–5.11	b = −1.17, *p =* 0.76, r = −0.02, CI = −8.76–6.41	–	–	–	–	b = −3.88, *p =* 0.35, r = −0.07, CI = −12.06–4.30	b = 0.73, *p =* 0.66, r = 0.03, CI = −2.56–4.02	–	–	–	–
*>1 SCI per decade (all, n = 154)*	b = −0.62, *p =* 0.88, r = −0.01, CI = −8.59–7.35	–	–	–	–	–	–	–	–	–	–	–	–	–	–
*Any SCI* *(SCA only, n = 106)*	b = −1.88, *p =* 0.52, r = −0.06, CI = −7.69–3.92	–	–	b = −0.92, *p =* 0.81, r = −0.02, CI = −8.44–6.59	b = −1.55, *p =* 0.69, r = −0.04, CI = −9.21–6.11	–	–	–	–	b = −3.72, *p =* 0.39, r = −0.08, CI = −12.27–4.83	b = 0.96, *p =* 0.56, r = 0.05, CI = −2.32–4.24	–	–	–	–
**CI**
***L-SCI*** *any SCI* *(all, n = 154)*	OR = 1.00, *p =* 0.99, CI = 0.26–3.87	–	–	OR = 0.78, *p =* 0.81, CI = 0.10–6.19	OR = 1.20, *p =* 0.75, CI = 0.39–3.73	–	–	–	–	–	OR = 1.06, *p =* 0.69, CI = 0.81–1.38	–	–	–	–
*>1 SCI per decade (all, n = 154)*	OR = 1.49, *p =* 0.57, CI = 0.39–5.74	–	–	–	–	–	–	–	–	–	–	–	–	–	–
*Any SCI* *(SCA only, n = 106)*	OR = 0.63, *p =* 0.55, CI = 0.14–2.87	–	–	OR = 0.45, *p =* 0.51, CI = 0.04–4.73	OR = 1.30, *p =* 0.70, CI = 0.35–4.80	–	–	–	–	–	OR = 0.98, *p =* 0.91, CI = 0.73–1.33	–	–	–	–
***T-SCI*** *any SCI* *(all, n = 154)*	OR = 1.28, *p =* 0.72, CI = 0.33–4.91	–	–	OR = 1.24, *p =* 0.82, CI = 0.20–7.87	OR = 1.03, *p =* 0.96, CI = 0.34–3.08	–	–	–	–	–	b = 1.08, *p =* 0.57, CI = 0.83–1.40	–	–	–	–
*>1 SCI per decade (all, n = 154)*	OR = 0.97, *p =* 0.96, CI = 0.23–4.00	–	–	–	–	–	–	–	–	–	–	–	–	–	–
*Any SCI* *(SCA only, n = 106)*	OR = 0.68, *p =* 0.63, CI = 0.15–3.16	–	–	OR = 0.57, *p =* 0.59, CI = 0.08–4.40	OR = 1.17, *p =* 0.79, CI = 0.35–3.91	–	–	–	–	–	OR = 0.99, *p =* 0.94, CI = 0.73–1.34	–	–	–	–
***R-SCI*** *any SCI* *(all, n = 154)*	OR = 2.02, *p =* 0.33, CI = 0.49–8.26	–	–	OR = 2.59, *p =* 0.27, CI = 0.48–14.17	OR = 0.59, *p =* 0.63, CI = 0.07–5.05	–	–	–	–	OR = 4.35, *p =* 0.13, CI = 0.65–29.16	OR = 0.58, *p =* 0.30, CI = 0.21–1.61	–	–	–	–
*>1 SCI per decade (all, n = 154)*	OR = 1.63, *p =* 0.64, CI = 0.21–12.61	–	–	–	–	–	–	–	–	–	–	–	–	–	–
*Any SCI* *(SCA only, n = 106)*	OR = 1.15, *p =* 0.86, CI = 0.24–5.51	–	–	OR = 1.44, *p =* 0.71, CI = 0.21–9.70	OR = 0.62, *p =* 0.69, CI = 0.06–6.57	–	–	–	–	OR = 2.51, *p =* 0.38, CI = 0.32–19.69	OR = 0.58, *p =* 0.32, CI = 0.20–1.69	–	–	–	–

*Values are regression coefficients (b), odds ratios (OR), probability values (p), semi-partial correlation coefficients (r), and 95% confidence intervals (CI) from global regression models. SCI, silent cerebral infarction; SCA, sickle cell anemia; No, number; Vol, volume; L-/T-/R-SCI, Liberal/Traditional/Restrictive silent cerebral infarction definitions*.

## References

[1] DeBaun MR Armstrong FD McKinstry RC Ware RE Vichinsky E Kirkham FJ. Silent cerebral infarcts: a review on a prevalent and progressive cause of neurologic injury in sickle cell anemia. Blood. (2012) 119:4587–96. 10.1182/blood-2011-02-272682 22354000 PMC3367871

[2] Casella JF King AA Barton B White DA Noetzel MJ Ichord RN. Design of the silent cerebral infarct transfusion (SIT) trial. Pediatr Hematol Oncol. (2010) 27:69–89. 10.3109/08880010903360367 20201689 PMC5572477

[3] DeBaun MR Gordon M McKinstry RC Noetzel MJ White DA Sarnaik SA. Controlled trial of transfusions for silent cerebral infarcts in sickle cell anemia. N Engl J Med. (2014) 371:699–710. 10.1056/NEJMoa1401731 25140956 PMC4195437

[4] Guilliams KP Fields ME Ragan DK Chen Y Eldeniz C Hulbert ML. Large-vessel vasculopathy in children with sickle cell disease: a magnetic resonance imaging study of infarct topography and focal atrophy. Pediatr Neurol. (2017) 69:49–57. 10.1016/j.pediatrneurol.2016.11.005 28159432 PMC5365370

[5] Kwiatkowski JL Zimmerman RA Pollock AN Seto W Smith-Whitley K Shults J. Silent infarcts in young children with sickle cell disease. Br J Haematol. (2009) 146:300–5. 10.1111/j.1365-2141.2009.07753.x 19500105 PMC2793684

[6] Cancio MI Helton KJ Schreiber JE Smeltzer MP Kang G Wang WC. Silent cerebral infarcts in very young children with sickle cell anaemia are associated with a higher risk of stroke. Br J Haematol. (2015) 171:120–9. 10.1111/bjh.13525 26058476

[7] Pegelow CH Macklin EA Moser FG Wang WC Bello JA Miller ST. Longitudinal changes in brain magnetic resonance imaging findings in children with sickle cell disease. Blood. (2002) 99:3014–18. 10.1182/blood.V99.8.3014 11929794

[8] Bernaudin F Verlhac S Arnaud C Kamdem A Vasile M Kasbi F. Chronic and acute anemia and extracranial internal carotid stenosis are risk factors for silent cerebral infarcts in sickle cell anemia. Blood. (2015) 125:1653–61. 10.1182/blood-2014-09-599852 25533032

[9] Kassim AA Pruthi S Day M Rodeghier M Gindville MC Brodsky MA. Silent cerebral infarcts and cerebral aneurysms are prevalent in adults with sickle cell anemia. Blood. (2016) 127:2038–40. 10.1182/blood-2016-01-694562 26941400

[10] Prussien KV Jordan LC DeBaun MR Compas BE. Cognitive function in sickle cell disease across domains, cerebral infarct status, and the lifespan: a meta-analysis. J Pediatr Psychol. (2019) 44:948–58. 10.1093/jpepsy/jsz031 31050352 PMC6706005

[11] Schatz J Brown RT Pascual JM Hsu L DeBaun MR. Poor school and cognitive functioning with silent cerebral infarcts and sickle cell disease. Neurology. (2001) 56:1109–11. 10.1212/WNL.56.8.1109 11320190

[12] DeBaun MR Jordan LC King AA Schatz J Vichinsky E Fox CK. American Society of Hematology 2020 guidelines for sickle cell disease: prevention, diagnosis, and treatment of cerebrovascular disease in children and adults. Blood Adv. (2020) 4:1554–88. 10.1182/bloodadvances.2019001142 32298430 PMC7189278

[13] Farrell AT Panepinto J Carroll CP Darbari DS Desai AA King AA. End points for sickle cell disease clinical trials: patient-reported outcomes, pain, the brain. Blood Adv. (2019) 3:3982–4001. 10.1182/bloodadvances.2019000882 31809538 PMC6963237

[14] Hijmans CT Grootenhuis MA Oosterlaan J Heijboer H Peters M Fijnvandraat K. Neurocognitive deficits in children with sickle cell disease are associated with the severity of anemia. Pediatr Blood Cancer. (2011) 57:297–302. 10.1002/pbc.22892 21671366

[15] Choi S O'Neil SH Joshi AA Li J Bush AM Coates TD. Anemia predicts lower white matter volume and cognitive performance in sickle and non-sickle cell anemia syndrome. Am J Hematol. (2019) 94:1055–65. 10.1002/ajh.25570 31259431 PMC6857783

[16] Stotesbury H Kirkham FJ Kölbel M Balfour P Clayden JD Sahota S. White matter integrity and processing speed in sickle cell anemia. Neurology. (2018) 90:e2042–50. 10.1212/WNL.0000000000005644 29752305 PMC5993179

[17] Vichinsky EP Neumayr LD Gold JI Weiner MW Rule RR Truran D. Neuropsychological dysfunction and neuroimaging abnormalities in neurologically intact adults with sickle cell anemia. JAMA. (2010) 303:1823–31. 10.1001/jama.2010.562 20460621 PMC2892214

[18] Choudhury NA DeBaun MR Rodeghier M King AA Strouse JJ McKinstry RC. Silent cerebral infarct definitions and full-scale IQ loss in children with sickle cell anemia. Neurology. (2018) 90:e239–46. 10.1212/WNL.0000000000004832 29263226 PMC5772160

[19] Kugler S Anderson B Cross D Sharif Z Sano M Haggerty R. Abnormal cranial magnetic resonance imaging scans in sickle-cell disease. Arch Neurol. (1993) 50:629. 10.1001/archneur.1993.00540060059019 8503800

[20] Bernaudin F Verlhac S Fréard F Roudot-Thoraval F Benkerrou M Thuret I. Multicenter prospective study of children with sickle cell disease: radiographic and psychometric correlation. J Child Neurol. (2000) 15:333–43. 10.1177/088307380001500510 10830200

[21] Houwing ME Grohssteiner RL Dremmen MHG Atiq F Bramer WM de Pagter APJ. Silent cerebral infarcts in patients with sickle cell disease: a systematic review and meta-analysis. BMC Med. (2020) 18:393. 10.1186/s12916-020-01864-8 33349253 PMC7754589

[22] van der Land V Zwanenburg JJM Fijnvandraat K Biemond BJ Hendrikse J Mutsaerts JMM. Cerebral lesions on 7 tesla MRI in patients with sickle cell anemia. Cerebrovasc Dis. (2015) 39:181–9. 10.1159/000373917 25765995

[23] Nelson MD Wilson DA Kisker CT Evatt BL Fenstermacher MJ Lynn HS. Incidence of focal white matter lesions in a population of hemophiliac children and their normal siblings. Pediatr Radiol. (2000) 30:705–9. 10.1007/s002470000290 11075607

[24] Brugulat-Serrat A Salvadó G Sudre CH Grau-Rivera O Suárez-Calvet M Falcon C. Patterns of white matter hyperintensities associated with cognition in middle-aged cognitively healthy individuals. Brain Imaging Behav. (2020) 14:2012–23. 10.1007/s11682-019-00151-2 31278650 PMC7572336

[25] Biesbroek JM Weaver NA Biessels GJ. Lesion location and cognitive impact of cerebral small vessel disease. Clin Sci. (2017) 131:715–28. 10.1042/CS20160452 28385827

[26] Choi S Bush AM Borzage MT Joshi AA Mack WJ Coates TD. Hemoglobin and mean platelet volume predicts diffuse T1-MRI white matter volume decrease in sickle cell disease patients. Neuroimage Clin. (2017) 15:239–46. 10.1016/j.nicl.2017.04.023 28540180 PMC5430155

[27] Schatz J Craft S Koby M Siegel MJ Resar L Lee RR. Neuropsychologic deficits in children with sickle cell disease and cerebral infarction: role of lesion site and volume. Child Neuropsychol. (1999) 5:92–103. 10.1076/chin.5.2.92.3170

[28] Schatz J White DA Moinuddin A Armstrong M DeBaun MR. Lesion burden and cognitive morbidity in children with sickle cell disease. J Child Neurol. (2002) 17:891–5. 10.1177/08830738020170122401 12593461

[29] van der Land V Hijmans CT de Ruiter M Mutsaerts HJMM Cnossen MH Engelen M. Volume of white matter hyperintensities is an independent predictor of intelligence quotient and processing speed in children with sickle cell disease. Br J Haematol. (2015) 168:553–6. 10.1111/bjh.13179 25303108

[30] Estcourt LJ Fortin PM Hopewell S Trivella M Doree C Abboud MR. Interventions for preventing silent cerebral infarcts in people with sickle cell disease. Cochrane Database Syst Rev. (2017) 5:CD012389. 10.1002/14651858.CD012389.pub2 28500860 PMC5460750

[31] Howard J Slee AE Skene S Inusa B Kawadler J Downes M. Overnight auto-adjusting continuous airway pressure + standard care compared with standard care alone in the prevention of morbidity in sickle cell disease phase II (POMS2b): study protocol for a randomised controlled trial. Trials. (2018) 19:55. 10.1186/s13063-017-2419-0 29357947 PMC5778753

[32] Rosen CL Debaun MR Strunk RC Redline S Seicean S Craven DI. Obstructive sleep apnea and sickle cell anemia. Pediatrics. (2014) 134:273–81. 10.1542/peds.2013-4223 25022740 PMC4187233

[33] Kölbel M Kirkham FJ Iles R Stotesbury H Halstead E Brenchley C. Exploring the relationship of sleep, cognition, and cortisol in sickle cell disease. Comprehens Psychoneuroendocrinol. (2022) 10:100128. 10.1016/j.cpnec.2022.100128 PMC9216257 35755206

[34] Wechsler D WASI-II. Wechsler abbreviated scale of intelligence - second edition. J Psychoeduc Assess. (2013) 31:337–41. 10.1177/0734282912467756

[35] Wechsler D. The Wechsler Intelligence Scale for Children. 4th ed. San Antonio, TX: The Psychological Corporation (2004).

[36] Wechsler D. Wechsler Adult Intelligence Scale. 4th ed (WAIS-IV). San Antonio, CA: The Psychological Corporation (2008). p. 1–3.

[37] DCLG. The English Indices of Deprivation 2015 Statistical Release (2015).

[38] Sudre CH Gomez Anson B Davagnanam I Schmitt A Mendelson AF Prados F. Bullseye's representation of cerebral white matter hyperintensities. J Neuroradiol. (2018) 45:114–22. 10.1016/j.neurad.2017.10.001 29132940 PMC5867449

[39] Fox J Weisberg S Adler D Bates D Baud-Bovy G Ellison S. car: Companion to Applied Regression. R Packag. Version 2.0-21, 1–157. California; London; New Delhi; Singapore: SAGE publications (2014).

[40] Peña EA Slate EH. Global validation of linear model assumptions. J Am Stat Assoc. (2006) 101:341–54. 10.1198/016214505000000637 20157621 PMC2820257

[41] Cohen J. Statistical Power Analysis for the Behavioral Sciences. 2nd ed. New York, NY: New York Academic Press (1988).

[42] King AA Strouse JJ Rodeghier MJ Compas BE Casella JF McKinstry RC. Parent education and biologic factors influence on cognition in sickle cell anemia. Am J Hematol. (2014) 89:162–7. 10.1002/ajh.23604 24123128 PMC4310566

[43] Choi E Branch C Cui MH Yazdanbakhsh K Mohandas N Billett HH. No evidence for cell activation or brain vaso-occlusion with plerixafor mobilization in sickle cell mice. Blood Cells Mol Dis. (2016) 57:67–70. 10.1016/j.bcmd.2015.12.008 26852658 PMC4806642

[44] Silva GS Vicari P Figueiredo MS Carrete H Jr. Idagawa MH Massaro AR. Brain magnetic resonance imaging abnormalities in adult patients with sickle cell disease: correlation with transcranial Doppler findings. Stroke. (2009) 40:2408–12. 10.1161/STROKEAHA.108.537415 19443807

[45] Wardlaw JM Smith EE Biessels GJ Cordonnier C Fazekas F Frayne R. Neuroimaging standards for research into small vessel disease and its contribution to ageing and neurodegeneration. Lancet Neurol. (2013) 12:822–38. 10.1016/S1474-4422(13)70124-8 23867200 PMC3714437

[46] Garnier-Crussard A Cotton F Desestret V Krolak-Salmon P Fort R. Silent cerebral infarcts in sickle cell disease: Really silent? J Neuroradiol. (2021) 48:471–2. 10.1016/j.neurad.2021.03.002 33727022

[47] Griffanti L Zamboni G Khan A Li L Bonifacio G Sundaresan V. BIANCA (Brain Intensity AbNormality Classification Algorithm): a new tool for automated segmentation of white matter hyperintensities. Neuroimage. (2016) 141:191–205. 10.1016/j.neuroimage.2016.07.018 27402600 PMC5035138

[48] Farris N Billett HH Branch CA Minniti C Branch K Acharya SA. The Association of white matter hyperintensity volume and sickle cell anemia. Blood. (2016) 128:1321. 10.1182/blood.V128.22.1321.132127432877

[49] Krejza J Arkuszewski M Radcliffe J Flynn TB Chen R Kwiatkowski JL. Association of pulsatility index in the middle cerebral artery with intelligence quotient in children with sickle cell disease. Neuroradiol J. (2012) 25:351–9. 10.1177/197140091202500311 24028989

[50] Chen R Krejza J Arkuszewski M Zimmerman RA Herskovits EH Melhem ER. Brain morphometric analysis predicts decline of intelligence quotient in children with sickle cell disease: a preliminary study. Adv Med Sci. (2017) 62:151–7. 10.1016/j.advms.2016.09.002 28279885 PMC5420463

[51] Downes M Keenan L Duane Y Duffy K Fortune G Geoghegan R. Executive function in children with sickle cell anemia on transfusion: NIH toolbox utility in the clinical context. Clin Neuropsychol. (2020) 1–16. 10.1080/13854046.2020.1847325. [Epub ahead of print]. 33200651

[52] Hood AM King AA Fields ME Ford AL Guilliams KP Hulbert ML. Higher executive abilities following a blood transfusion in children and young adults with sickle cell disease. Pediatr Blood Cancer. (2019) 66:e27899. 10.1002/pbc.27899 31267645 PMC6707832

[53] Farris N Branch CA Zimmerman ME Suri AK Billett HH. Lack of association of CNS lesion number with cognitive performance and cerebral blood flow in sickle cell disease. Blood. (2015) 126:984. 10.1182/blood.V126.23.984.984

[54] Sanger M Jordan L Pruthi S Day M Covert B Merriweather B. Cognitive deficits are associated with unemployment in adults with sickle cell anemia. J Clin Exp Neuropsychol. (2016) 38:661–71. 10.1080/13803395.2016.1149153 27167865

[55] Baldeweg T Hogan AM Saunders DE Telfer P Gadian DG Vargha-Khadem F. Detecting white matter injury in sickle cell disease using voxel-based morphometry. Ann Neurol. (2006) 59:662–72. 10.1002/ana.20790 16450382

[56] Wang W Enos L Gallagher D Thompson R Guarini L Vichinsky E. Neuropsychologic performance in school-aged children with sickle cell disease: a report from the Cooperative Study of Sickle Cell Disease. J Pediatr. (2001) 139:391–7. 10.1067/mpd.2001.116935 11562619

[57] Coloigner J Kim Y Bush A Choi S Balderrama MC Coates TD. Contrasting resting-state fMRI abnormalities from sickle and non-sickle anemia. PLoS ONE. (2017) 12:e0184860. 10.1371/journal.pone.0184860 28981541 PMC5628803

[58] Václavu L Petr J Petersen ET Mutsaerts HJMM Majoie CBL Wood JC. Cerebral oxygen metabolism in adults with sickle cell disease. Am J Hematol. (2020) 95:401–12. 10.1002/ajh.25727 31919876 PMC7155077

[59] Chai Y Ji C Coloigner J Choi S Balderrama M Vu C. Tract-specific analysis and neurocognitive functioning in sickle cell patients without history of overt stroke. Brain Behav. (2021) 11:e01978. 10.1002/brb3.1978 33434353 PMC7994688

[60] Wang WC Zou P Hwang SN Kang G Ding J Heitzer AM. Effects of hydroxyurea on brain function in children with sickle cell anemia. Pediatr Blood Cancer. (2021) 68:e29254. 10.1002/pbc.29254 34331507

[61] Heitzer AM Longoria J Okhomina V Wang WC Raches D Potter B. Hydroxyurea treatment and neurocognitive functioning in sickle cell disease from school age to young adulthood. Br J Haematol. (2021) 195:256–66. 10.1111/bjh.17687 34272726 PMC8900674

[62] Wang Y Fellah S Fields ME Guilliams KP Binkley MM Eldeniz C. Cerebral oxygen metabolic stress, microstructural injury, and infarction in adults with sickle cell disease. Neurology. (2021) 97:e902–12. 10.1212/WNL.0000000000012404 34172536 PMC8408504

[63] Fields ME Guilliams KP Ragan DK Binkley MM Eldeniz C Chen Y. Regional oxygen extraction predicts border zone vulnerability to stroke in sickle cell disease. Neurology. (2018) 90:e1134–42. 10.1212/WNL.0000000000005194 29500287 PMC5880632

[64] Ford AL Ragan DK Fellah S Binkley MM Fields ME Guilliams KP. Silent infarcts in sickle cell disease occur in the border zone region and are associated with low cerebral blood flow. Blood. (2018) 132:1714–23. 10.1182/blood-2018-04-841247 30061156 PMC6194388

[65] Stotesbury H Hales PW Hood AM Koelbel M Kawadler JM Saunders DE. (2022). Individual watershed areas in sickle cell anemia: An arterial spin labeling study. Front Physiol. 13:865391. 10.3389/fphys.2022.865391 35592036 PMC9110791

[66] Kim KW MacFall JR Payne ME. Classification of white matter lesions on magnetic resonance imaging in elderly persons. Biol Psychiatry. (2008) 64:273–80. 10.1016/j.biopsych.2008.03.024 18471801 PMC2593803

[67] Stotesbury H Kawadler JM Hales PW Saunders DE Clark CA Kirkham FJ. Vascular instability and neurological morbidity in sickle cell disease: an integrative framework. Front Neurol. (2019) 10:871. 10.3389/fneur.2019.00871 31474929 PMC6705232

[68] Miao X Choi S Tamrazi B Chai Y Vu C Coates TD. Increased brain iron deposition in patients with sickle cell disease: an MRI, quantitative susceptibility mapping study. Blood. (2018) 132:1618–21. 10.1182/blood-2018-04-840322 30045839 PMC6182265

[69] Kirkham FJ Shmueli K. Brain iron in sickle cell disease? Blood. (2018) 132:1550–2. 10.1182/blood-2018-08-867010 30309876

[70] Kawadler JM Kirkham FJ Clayden JD Hollocks MJ Seymour EL Edey R. White matter damage relates to oxygen saturation in children with sickle cell anemia without silent cerebral infarcts. Stroke. (2015) 46:1793–9. 10.1161/STROKEAHA.115.008721 25967572

[71] Stotesbury H Kawadler JM Saunders DE Kirkham FJ. MRI detection of brain abnormality in sickle cell disease. Expert Rev Hematol. (2021) 14:473–91. 10.1080/17474086.2021.1893687 33612034 PMC8315209

[72] Stotesbury H Hales PW Koelbel M Hood AM Kawadler JM Saunders DE. Venous cerebral blood flow quantification and cognition in patients with sickle cell anemia. J Cereb Blood Flow Metab. (2022) 271678X211072391. 10.1177/0271678X211072391. [Epub ahead of print]. 34986673 PMC9121533

[73] Prussien KV Compas BE Siciliano RE Ciriegio AE Lee CA Kassim AA. Cerebral hemodynamics, and executive function in sickle cell, anemia. Stroke. (2021) 52:1830–4. 10.1161/STROKEAHA.120.032741 33840223 PMC8483619

[74] Fields ME Mirro AE Guilliams KP Binkley MM Gil Diaz L Tan J. Functional connectivity decreases with metabolic stress in sickle cell disease. Ann Neurol. (2020) 88:995–1008. 10.1002/ana.25891 32869335 PMC7592195

[75] Kawadler JM Clayden JD Kirkham FJ Cox TC Saunders DE Clark CA. Subcortical and cerebellar volumetric deficits in paediatric sickle cell anaemia. Br J Haematol. (2013) 163:373–6. 10.1111/bjh.12496 23889205

[76] Santini T Koo M Farhat N Campos VP Alkhateeb S Vieira MAC. Analysis of hippocampal subfields in sickle cell disease using ultrahigh field MRI. Neuroimage Clin. (2021) 30:102655. 10.1016/j.nicl.2021.102655 34215139 PMC8102634

[77] Dowling MM Quinn CT Plumb P Rogers ZR Rollins NK Koral K. Acute silent cerebral ischemia and infarction during acute anemia in children with and without sickle cell disease. Blood. (2012) 120:3891–7. 10.1182/blood-2012-01-406314 22948048 PMC3496951

