# Supplementary material for: Quantification of Silent Cerebral Infarction on High-Resolution FLAIR and Cognition in Sickle Cell Anemia
Source: Front Neurol. 2022 Jun 29;13:867329. doi: 10.3389/fneur.2022.867329 (PMC9277177; doi:10.3389/fneur.2022.867329)
Supplement: Supplementary file 1 [file Data_Sheet_1.docx]

Supplementary Material

Supplementary Table 1. Regional Regression Models………..………………………………………………………………….2

Supplementary Table 2 1.5T studies (defined or assumed based on year)………………………………………………………..7

Supplementary Figure 1. Correlations between predictors for models run in people with SCA only……………………………10

Supplementary Figure 2. Lesion distribution for traditionally-defined SCI (T-SCI)……………………………………………..11

Supplementary Figure 3. Lesion distribution for restrictively-defined SCI (L-SCI)……………………………………………..12

| **Regional Models** | **1a) Lobar SCI** | | **1b) Regional SCI** | | **2a) Lobar SCI + Lobar SCI Number** | | | | **2b) Regional SCI + Regional SCI Number** | | | | **3a) Lobar SCI + Lobar SCI Volume** | | | | **3b) Lobar SCI + Lobar SCI Volume** | | | |
| --- | --- | --- | --- | --- | --- | --- | --- | --- | --- | --- | --- | --- | --- | --- | --- | --- | --- | --- | --- | --- |
| *Predictor* | **SCI Fr.** | **SCI**  **Pa.** | **SCI**  **Dp.** | **SCI**  **Jc.** | **SCI**  **Fr.** | **SCI Fr.**  **No.** | **SCI**  **Pa.** | **SCI Pa. No.** | **SCI Dp.** | **SCI Dp. No.** | **SCI Jc.** | **SCI Jc. No.** | **SCI**  **Fr.** | **SCI Fr.**  **Vol.** | **SCI**  **Pa.** | **SCI Pa. Vol.** | **SCI Dp.** | **SCI Dp. Vol.** | **SCI Jc.** | **SCI Jc. Vol.** |
| **FSIQ** |  |  |  |  |  |  |  |  |  |  |  |  |  |  |  |  |  |  |  |  |
| ***L-SCI***  *all*  *(n=154)* | b=-1.83, p=0.47, r=-0.06, CI=  -6.80 –3.15 | b=0.67, p=0.83, r=0.02, CI=  -5.49 –  6.82 | b=-2.30, p=0.39, r=-0.07, CI=  -7.58 –2.97 | b=0.37, p=0.90, r=0.01, CI=  -5.72 –  6.46 | b=-2.11, p=0.52, r=-0.05, CI=  -8.56 –  4.35 | b=1.25, p=0.63, r=0.04, CI=  -3.84 –  6.33 | b=4.82, p=0.33, r=0.08, CI=  -4.86 –14.50 | b=-4.97, p=0.25, r=-0.09, CI=  -13.56 –3.62 | b=-2.96, p=0.36, r=-0.07, CI=  -9.32 –  3.39 | b=1.06, p=0.62, r=0.04, CI=  -3.17 –  5.28 | b=2.07, p=0.62, r=0.04, CI=  -6.20 –10.33 | b=-2.28, p=0.50, r=-0.05, CI=  -9.01 –  4.44 | - | b=-0.01, p=0.99, r=-0.00, CI=  -1.38 –  1.36 |  | b=-0.45, p=0.56, r=-0.05, CI=  -1.96 –  1.06 | - | b=0.25, p=0.78, r=0.02, CI=  -1.52 –  2.01 | - | b=-0.83, p=0.45, r=-0.06, CI=  -2.98 –1.32 |
| *SCA (n=106)* | b=1.42, p=0.64, r=0.04, CI=  -4.57 –  7.41 | b=-3.12, p=0.42, r=-0.08, CI=  -10.74 – 4.50 | b=-2.36, p=0.45, r=-0.07, CI=  -8.50 –  3.78 | b=1.61, p=0.65, r=0.04, CI=  -5.48 –  8.70 | – | b=1.72, p=0.46, r=0.07, CI=  -2.87 –  6.30 | – | b=-4.16, p=0.22, r=-0.12, CI=  -10.81 –2.49 | b=-2.74, p=0.49, r=-0.07, CI=  -10.54 –5.06 | b=0.67, p=0.79, r=0.03, CI=  -4.23 –  5.57 | b=3.74, p=0.43, r=0.08,  CI=  -5.65 –  13.13 | b=-2.78, p=0.47, r=-0.07, CI=  -10.36 –4.80 | - | b=0.65, p=0.40, r=0.08, CI=  -0.88 –  2.18 | - | b=-1.18, p=0.17, r=-0.13, CI=  -2.87 –  0.52 | - | b=0.19, p=0.85, r=0.02, CI=  -1.81 –  2.18 | - | b=-0.65, p=0.60, r=-0.05, CI=  -3.08 –1.78 |
| ***T-SCI***  *all*  *(n=154)* | b=-0.75, p=0.78, r=-0.02, CI=  -6.15 –4.65 | b=0.22, p=0.95, r=0.00, CI=  -6.63 –  7.07 | b=-0.64, p=0.83, r=-0.02, CI=  -6.41 –5.12 | b=-0.20, p=0.95, r=-0.00, CI=  -6.85–  6.44 | b=-2.07, p=0.55, r=-0.05, CI=  -8.91–  4.77 | b=2.36, p=0.42, r=0.06, CI=  -3.44 –  8.17 | b=4.24, p=0.38, r=0.07, CI=  -5.21 –13.69 | b=-6.42, p=0.18, r=-0.11, CI=  -15.84 –3.00 | b=-0.51, p=0.88, r=-0.01, CI=  -7.44 –  6.41 | b=-0.12, p=0.96, r=-0.00, CI=  -4.91 –  4.68 | b=0.33, p=0.94, r=0.01, CI=  -7.88 –8.53 | b=-0.79, p=0.84, r=-0.02, CI=  -8.56 –  6.98 |  | b=0.16, p=0.82, r=0.02, CI=  -1.23 –  1.55 |  | b=-0.55, p=0.49, r=-0.05, CI=  -2.14 –  1.04 |  | b=0.06, p=0.95, r=0.01, CI=  -1.77 –  1.89 |  | b=-0.45, p=0.70, r=-0.03, CI=  -2.73 –1.83 |
| *SCA*  *(n=106)* | b=2.88, p=0.34, r=0.09, CI=  -3.12 –8.89 | b=-3.73, p=0.35, r=-0.09, CI=  -11.55 –4.08 | b=-0.88, p=0.78, r=-0.03, CI=  -7.30 –5.53 | b=1.14, p=0.76, r=0.03, CI=  -6.16 –  8.43 | b=2.67, p=0.48, r=0.07, CI=  -4.78 –10.12 | b=0.81, p=0.79, r=0.02, CI=  -5.27 –  6.89 | b=0.43, p=0.94, r=0.01, CI=-10.94 –11.81 | b=-5.14, p=0.33, r=-0.09, CI=  -15.47 –5.19 | b=-0.44, p=0.91, r=-0.01, CI=  -8.26 –  7.39 | b=-0.45, p=0.86, r=-0.02, CI=  -5.47 –  4.58 | b=1.80, p=0.69, r=0.04, CI=  -7.09 –10.68 | b=-0.91, p=0.82, r=-0.02, CI=  -9.04 –  7.22 | - | b=0.73, p=0.34, r=0.09, CI=  -0.77 –  2.24 | - | b=-1.20, p=0.17, r=-0.13, CI=  -2.90 – 0.51 | - | b=-0.05, p=0.96, r=-0.00, CI=  -1.99 –  1.90 | - | b=-0.21, p=0.86, r=-0.02, CI=  -2.62 –2.19 |
| ***R-SCI***  all  (n=154) | b=1.22, p=0.70, r=0.03, CI=  -5.07 –7.51 | b=-3.83, p=0.49, r=-0.06, CI=  -14.74 –7.08 | b=-0.98, p=0.76, r=-0.02, CI=  -7.24 –5.28 | b=0.64, p=0.91, r=0.01, CI=  -11.03 –12.30 | b=-1.99, p=0.60, r=-0.04, CI=  -9.50 –  5.52 | b=6.51, p=0.19, r=0.10, CI=  -3.19 –16.21 | b=-0.39, p=0.96, r=-0.00, CI=  -14.96 –14.19 | b=-23.21, p=0.13, r=-0.12, CI=  -53.00 –6.59 | b=-2.45, p=0.55, r=-0.05, CI=  -10.51 –5.61 | b=2.58, p=0.56, r=0.05, CI=  -6.25 –11.41 | b=-2.02, p=0.82, r=-0.02, CI=  -19.10 –15.05 | b=4.65, p=0.68, r=0.03, CI=  -17.60 –26.91 | b=-2.87, p=0.52, r=-0.05, CI=  -11.61 –5.87 | b=3.03, p=0.14, r=0.12, CI=  -1.00 –  7.06 | b=2.96, p=0.74, r=0.03, CI=  -14.84 –20.75 | b=-5.61, p=0.14, r=-0.12, CI=  -13.11 –1.89 | b=-4.14, p=0.31, r=-0.08, CI=  -12.12 – 3.85 | b=3.22, p=0.11, r=0.13, CI=  -0.69 –  7.13 | b=4.08, p=0.58, r=0.04, CI=  -10.41 – 18.57 | b=-4.08, p=0.27, r=-0.09, CI=  -11.43 – 3.26 |
| *SCA*  *(n=106)* | b=2.50, p=0.46, r=0.07, CI=  -4.27 –9.27 | b=-6.31, p=0.31, r=-0.10, CI=  -18.52 –5.90 | b=-0.61, p=0.85, r=-0.02, CI=  -7.24 –  6.01 | b=0.17, p=0.98, r=0.00, CI=  -11.72 –  12.06 | b=-0.21, p=0.96, r=-0.00, CI=  -8.16 –  7.73 | b=5.00, p=0.33, r=0.09, CI=  -5.17 –15.18 | b=-1.42, p=0.87, r=-0.01, CI=  -19.23 –16.39 | b=-23.06, p=0.16, r=-0.13, CI=  -55.75 –9.62 | b=-1.46, p=0.74, r=-0.03, CI=  -10.20 –7.29 | b=1.37, p=0.77, r=0.03, CI=  -7.87 –10.61 | b=-2.61, p=0.77, r=-0.03, CI=  -20.14 –14.91 | b=4.92, p=0.67, r=0.04, CI=  -17.77 –27.62 | b=-1.48, p=0.75, r=-0.03, CI=  -10.54 –7.59 | b=2.85, p=0.18, r=0.12, CI=  -1.37 –  7.07 | b=-1.49, p=0.89, r=-0.01, CI=  -23.06 –20.07 | b=-4.20, p=0.31, r=-0.09, CI=  -12.41 –4.01 | b=-4.09, p=0.35, r=-0.09, CI=  -12.64 – 4.47 | b=3.36, p=0.11, r=0.15, CI=  -0.79 –  7.51 | b=4.33, p=0.57, r=0.05, CI=  -10.59 –19.25 | b=-4.50, p=0.25, r=-0.11, CI=  -12.15 – 3.15 |
| **Supplementary Table 1. Regional Regression Models.** Values are regression coefficients (b), odds ratios (OR), probability values (p), semi-partial correlation coefficients (r), and 95% confidence intervals (CI) from regional regression models. Abbreviations; SCI=silent cerebral infarction, SCA=sickle cell anemia , No=number, Vol=volume, L-/T-/R-SCI = Liberal/Traditional/Restrictive silent cerebral infarction definitions | | | | | | | | | | | | | | | | | | | | |

| **Regional Models** | **1a) Lobar SCI** | | **1b) Regional SCI** | | **2a) Lobar SCI + Lobar SCI Number** | | | | **2b) Regional SCI + Regional SCI Number** | | | | **3a) Lobar SCI + Lobar SCI Volume** | | | | **3b) Lobar SCI + Lobar SCI Volume** | | | |
| --- | --- | --- | --- | --- | --- | --- | --- | --- | --- | --- | --- | --- | --- | --- | --- | --- | --- | --- | --- | --- |
| *Predictor* | **SCI Fr.** | **SCI**  **Pa.** | **SCI**  **Dp.** | **SCI**  **Jc.** | **SCI**  **Fr.** | **SCI Fr.**  **No.** | **SCI**  **Pa.** | **SCI Pa. No.** | **SCI Dp.** | **SCI Dp. No.** | **SCI Jc.** | **SCI Jc. No.** | **SCI**  **Fr.** | **SCI Fr.**  **Vol.** | **SCI**  **Pa.** | **SCI Pa. Vol.** | **SCI Dp.** | **SCI Dp. Vol.** | **SCI Jc.** | **SCI Jc. Vol.** |
| **WMI** |  |  |  |  |  |  |  |  |  |  |  |  |  |  |  |  |  |  |  |  |
| ***L-SCI***  *all*  *(n=154)* | b=-0.19, p=0.95, r=-0.01, CI=  -5.64 –  5.27 | b=-0.58, p=0.87, r=-0.01, CI=  -7.32 –6.17 | b=-3.15, p=0.28, r=-0.09, CI=  -8.91 –2.62 | b=1.31, p=0.70, r=0.03, CI=  -5.35 –  7.96 | b=2.34, p=0.51, r=0.05,  CI=  -4.73 –  9.42 | b=-2.74, p=0.33, r=-0.08, CI=  -8.32 –  2.83 | b=1.24, p=0.82, r=0.02, CI=  -9.37 –11.85 | b=-0.49, p=0.92, r=-0.01, CI=  -9.91 –  8.93 | b=-3.32, p=0.35, r=-0.08, CI=  -10.27 –3.62 | b=0.58, p=0.81, r=0.02, CI=  -4.04 –  5.19 | b=3.67, p=0.42, r=0.06, CI=  -5.37 –12.70 | b=-2.92, p=0.43, r=-0.06, CI=  -10.27 –4.42 | – | b=-0.27, p=0.72, r=-0.03, CI=  -1.78 –  1.23 | – | b=-0.23, p=0.78, r=-0.02, CI=  -1.88 –  1.42 | – | b=0.08, p=0.93, r=0.01, CI=  -1.85 –2.01 | – | b=-0.70, p=0.56, r=-0.05, CI=  -3.06 –1.65 |
| *SCA (n=106)* | b=3.40, p=0.30, r=0.10, CI=  -3.10 –9.90 | b=-3.74, p=0.37, r=-0.09, CI=  -12.00 –4.53 | b=-2.43, p=0.47, r=-0.07, CI=  -9.11 –4.24 | b=2.12, p=0.59, r=0.05, CI=  -5.59 –  9.83 | – | b=-0.74, p=0.77, r=-0.03, CI=  -5.75 –  4.27 | – | b=-1.12, p=0.76, r=-0.03, CI=  -8.39 –  6.15 | b=-1.80, p=0.67, r=-0.04, CI=  -10.27 –6.67 | b=-0.22, p=0.93, r=-0.01, CI=  -5.54 –  5.10 | b=5.39, p=0.30, r=0.10, CI=  -4.80 –15.58 | b=-3.77, p=0.37, r=-0.09, CI=  -11.99 –4.46 | – | b=0.35, p=0.68, r=0.04, CI=  -1.32 –  2.02 | – | b=-0.87, p=0.36, r=-0.09, CI=  -2.73 –  0.99 | – | b=0.18, p=0.87, r=0.02, CI=  -1.99 –2.35 | – | b=-0.68, p=0.61, r=-0.05, CI=  -3.32 –1.97 |
| ***T-SCI***  *all*  *(n=154)* | b=0.03, p=0.99, r=0.00, CI=  -5.88 –5.93 | b=0.59, p=0.88, r=0.01, CI=  -6.90 –8.09 | b=-2.09, p=0.51, r=-0.05, CI=  -8.39 –4.21 | b=1.04, p=0.78, r=0.02, CI=  -6.22 –  8.31 | b=1.32, p=0.73, r=0.03,  CI=  -6.17-  8.81 | b=-1.17, p=0.72, r=-0.03, CI=  -7.53-  5.19 | b=4.82, p=0.36, r=0.07, CI=  -5.52-15.17 | b=-4.78, p=0.36, r=-0.07, CI=  -15.09-5.54 | b=-2.32, p=0.55, r=-0.05, CI=  -9.89 –  5.25 | b=0.39, p=0.88, r=0.01, CI=  -4.85 –  5.63 | b=2.28, p=0.62, r=0.04, CI=  -6.69 –11.24 | b=-2.03, p=0.64, r=-0.04, CI=  -10.53 –6.46 | – | b=-0.21, p=0.79, r=-0.02, CI=  -1.73 –  1.31 | – | b=-0.15, p=0.86, r=-0.01, CI=  -1.89 –  1.59 | – | b=0.23, p=0.82, r=0.02, CI=  -1.77 –2.23 | – | b=-0.73, p=0.56, r=-0.05, CI=  -3.23 –1.76 |
| *SCA*  *(n=106)* | b=3.61, p=0.28, r=0.10, CI=  -2.93 –10.14 | b=-3.06, p=0.48, r=-0.07, CI=  -11.56 –5.44 | b=-2.04, p=0.56, r=-0.06, CI=  -9.01 –4.93 | b=2.04, p=0.61, r=0.05, CI=  -5.88 –  9.97 | b=6.18, p=0.13, r=0.15,  CI=  -1.87 –  14.23 | b=-2.95, p=0.37, r=-0.09, CI=  -9.52 –  3.61 | b=1.85, p=0.77, r=0.03, CI=  -10.44 –14.14 | b=-3.74, p=0.51, r=-0.06, CI=  -14.90 –7.43 | b=-1.86, p=0.66, r=-0.04, CI=  -10.34 –6.62 | b=-0.04, p=0.99, r=-0.00, CI=  -5.49 –  5.41 | b=3.96, p=0.42, r=0.08, CI=  -5.68 –13.59 | b=-3.00, p=0.50, r=-0.07, CI=  -11.81 –5.82 | – | b=0.35, p=0.68, r=0.04, CI=  -1.31 –  2.00 | – | b=-0.77, p=0.42, r=-0.08, CI=  -2.64 –  1.10 | – | b=0.24, p=0.82, r=0.02, CI=  -1.88 –2.36 | – | b=-0.66, p=0.62, r=-0.05, CI=  -3.28 –1.95 |
| ***R-SCI***  all  (n=154) | b=-0.24, p=0.95, r=-0.01, CI=  -7.10 –6.62 | b=-6.60, p=0.28, r=-0.09, CI=  -18.50 –5.31 | b=-1.23, p=0.72, r=-0.03, CI=  -8.08 –5.62 | b=3.42, p=0.60, r=0.04, CI=  -9.33 –16.18 | b=-2.72, p=0.51,  r=-0.05,  CI=  -10.97 –  5.52 | b=4.98, p=0.36, r=0.07, CI=  -5.67 –15.64 | b=-3.55, p=0.66, r=-0.03, CI=  -19.56 –12.46 | b=-19.22, p=0.25, r=-0.09, CI=  -51.94 –13.50 | b=-0.90, p=0.84, r=-0.02, CI=  -9.68 –  7.88 | b=-0.56, p=0.91, r=-0.01, CI=  -10.18 –9.05 | b=12.23, p=0.20, r=0.10, CI=  -6.37 –30.82 | b=-15.76, p=0.20, r=-0.10, CI=  -39.99 –8.48 | b=-4.75, p=0.33, r=-0.08, CI=  -14.35 –4.85 | b=3.19, p=0.16, r=0.11, CI=  -1.24 –  7.62 | b=-3.95, p=0.69, r=-0.03, CI=  -23.51 –15.61 | b=-3.31, p=0.43, r=-0.06, CI=  -11.55 –4.94 | b=-2.74, p=0.54, r=-0.05, CI=  -11.54 –6.06 | b=1.28, p=0.56, r=0.05, CI=  -3.03 –5.59 | b=4.04, p=0.62, r=0.04, CI=  -11.92 –20.01 | b=-1.06, p=0.80, r=-0.02, CI=  -9.16 –7.03 |
| *SCA*  *(n=106)* | b=2.13, p=0.57, r=0.05, CI=  -5.19 –9.45 | b=-10.20, p=0.13, r=-0.15, CI=  -23.40 –3.01 | b=-0.83, p=0.82, r=-0.02, CI=  -8.03 –6.37 | b=3.52, p=0.59, r=0.05, CI=  -9.40 –16.44 | b=0.01, p=1.00, r=0.00,  CI=  -8.64 –  8.66 | b=3.78, p=0.50, r=0.06, CI=  -7.30 –14.86 | b=-5.63, p=0.57, r=-0.05, CI=  -25.02 –13.76 | b=-19.99, p=0.27, r=-0.11, CI=  -55.57 –15.60 | b=0.09, p=0.98, r=0.00, CI=  -9.33 –  9.52 | b=-1.48, p=0.77, r=-0.03, CI=  -11.43 –8.47 | b=12.67,  p=0.19, r=0.13, CI=  -6.21 –31.55 | b=-16.23, p=0.19, r=-0.13, CI=  -40.68 –8.22 | – | – | – | – | b=-2.93, p=0.54, r=-0.06, CI=  -12.32 –6.46 | b=1.87, p=0.42, r=0.08, CI=  -2.69 –6.42 | b=5.33, p=0.52, r=0.06, CI=  -11.04 –21.70 | b=-2.13, p=0.62, r=-0.05, CI=  -10.52 –6.27 |

**Supplementary Table 1. Cont.**

| **Regional Models** | **1a) Lobar SCI** | | **1b) Regional SCI** | | **2a) Lobar SCI + Lobar SCI Number** | | | | **2b) Regional SCI + Regional SCI Number** | | | | **3a) Lobar SCI + Lobar SCI Volume** | | | | **3b) Lobar SCI + Lobar SCI Volume** | | | |
| --- | --- | --- | --- | --- | --- | --- | --- | --- | --- | --- | --- | --- | --- | --- | --- | --- | --- | --- | --- | --- |
| *Predictor* | **SCI Fr.** | **SCI**  **Pa.** | **SCI**  **Dp.** | **SCI**  **Jc.** | **SCI**  **Fr.** | **SCI Fr.**  **No.** | **SCI**  **Pa.** | **SCI Pa. No.** | **SCI Dp.** | **SCI Dp. No.** | **SCI Jc.** | **SCI Jc. No.** | **SCI**  **Fr.** | **SCI Fr.**  **Vol.** | **SCI**  **Pa.** | **SCI Pa. Vol.** | **SCI Dp.** | **SCI Dp. Vol.** | **SCI Jc.** | **SCI Jc. Vol.** |
| **PSI** |  |  |  |  |  |  |  |  |  |  |  |  |  |  |  |  |  |  |  |  |
| ***L-SCI***  *all*  *(n=154)* | b=-1.61, p=0.52, r=-0.05, CI=  -6.58 –3.36 | b=-1.65, p=0.60, r=-0.04, CI=  -7.79 –4.50 | b=-0.90, p=0.74, r=-0.03, CI=  -6.17 –4.38 | b=-2.20, p=0.48, r=-0.05, CI=  -8.28 –  3.89 | b=-3.72, p=0.26, r=-0.09, CI=  -10.16 –2.72 | b=3.14, p=0.22, r=0.09, CI=  -1.94 –  8.21 | b=0.76, p=0.88, r=0.01, CI=  -8.90 –10.42 | b=-4.12, p=0.34, r=-0.07, CI=  -12.69 –4.46 | b=-1.05, p=0.75, r=-0.03, CI=  -7.41 –5.32 | b=0.08, p=0.97, r=0.00, CI=  -4.15 –  4.31 | b=-2.86, p=0.50, r=-0.05, CI=  -11.14 –5.42 | b=0.75, p=0.83, r=0.02, CI=  -5.98 –  7.49 | – | b=0.35, p=0.62, r=0.04, CI=  -1.02 –  1.72 | – | b=-1.06, p=0.16, r=-0.11, CI=  -2.57 –  0.44 | – | b=0.52, p=0.56, r=0.04, CI=  -1.24 –  2.28 | – | b=-1.39, p=0.20, r=-0.10, CI=  -3.53 –0.75 |
| *SCA (n=106)* | b=2.20, p=0.45, r=0.07, CI=  -3.52 –7.92 | b=-4.74, p=0.20, r=-0.12, CI=  -12.02 –2.54 | b=0.87, p=0.77, r=0.03, CI=  -5.02 –  6.77 | b=-2.25, p=0.51, r=-0.06, CI=  -9.05 –  4.56 | – | b=3.21, p=0.15, r=0.13, CI=  -1.15 –7.56 | – | b=-5.91, p=0.07, r=-0.17, CI=  -12.23 –0.41 | b=0.77, p=0.84, r=0.02, CI=  -6.74 –  8.27 | b=0.25, p=0.91, r=0.01, CI=  -4.46 –  4.97 | b=-1.16, p=0.80, r=-0.02, CI=  -10.19 –7.88 | b=-1.38, p=0.71, r=-0.04, CI=  -8.68 –  5.91 | – | b=1.19, p=0.11, r=0.15, CI=  -0.26 –  2.63 | – | **b=-1.76, p=0.03, r=-0.20, CI=**  **-3.36 –0.15** | – | b=1.02, p=0.29, r=0.10, CI=  -0.88 –  2.92 | – | b=-1.68, p=0.15, r=-0.13, CI=  -3.99 –0.63 |
| ***T-SCI***  *all*  *(n=154)* | b=-0.28, p=0.92, r=-0.01, CI=  -5.66 –5.11 | b=-2.94, p=0.40, r=-0.07, CI=  -9.77 –3.90 | b=1.25, p=0.67, r=0.03, CI=  -4.50 –6.99 | b=-3.87, p=0.25, r=-0.09, CI=  -10.49 –2.75 | b=-2.73, p=0.43, r=-0.06, CI=  -9.52 –  4.06 | b=3.98, p=0.17, r=0.10, CI=  -1.78 –  9.75 | b=1.67, p=0.73, r=0.03, CI=  -7.72 –11.05 | b=-8.05, p=0.09, r=-0.13, CI=  -17.40 –1.31 | b=1.80, p=0.61, r=0.04, CI=  -5.11 –8.70 | b=-0.68, p=0.78, r=-0.02, CI=  -5.45 –  4.10 | b=-3.70, p=0.37, r=-0.07, CI=  -11.87 –4.48 | b=-0.07, p=0.99, r=-0.00, CI=  -7.82 –  7.67 | – | b=0.44, p=0.53, r=0.05, CI=  -0.94 –  1.83 | – | b=-1.23, p=0.13, r=-0.12, CI=  -2.81 –  0.35 |  | b=0.83, p=0.37, r=0.07, CI=  -0.98 –  2.65 |  | b=-1.74, p=0.13, r=-0.12, CI=  -4.01 –0.52 |
| *SCA*  *(n=106)* | b=4.15, p=0.15, r=0.13, CI=  -1.54 –9.85 | b=-6.82, p=0.07, r=-0.17, CI=  -14.23 –0.58 | b=3.16, p=0.31, r=0.09, CI=  -2.94 –9.27 | b=-4.10, p=0.24, r=-0.11, CI=  -11.05 –2.85 | b=2.59, p=0.47, r=0.07, CI=  -4.42 –  9.59 | b=2.83, p=0.33, r=0.09, CI=  -2.88 –  8.54 | b=-1.46, p=0.79, r=-0.02, CI=  -12.15 –9.23 | b=-7.68, p=0.12, r=-0.14, CI=  -17.39 –2.03 | b=4.05, p=0.28, r=0.10, CI=  -3.40 –11.49 | b=-0.96, p=0.69, r=-0.04, CI=  -5.74 –  3.82 | b=-3.63, p=0.40, r=-0.08, CI=  -12.09 –4.83 | b=-0.47, p=0.90, r=-0.01, CI=  -8.21 –  7.27 | – | b=1.26, p=0.08, r=0.16, CI=  -0.16 –  2.68 | – | **b=-1.95, p=0.02, r=-0.22, CI=**  **-3.55 –**  **-0.34** |  | b=1.13, p=0.23, r=0.11, CI=  -0.72 –  2.97 |  | b=-1.83, p=0.11, r=-0.15, CI=  -4.11 –0.45 |
| ***R-SCI***  all  (n=154) | b=-0.54, p=0.86, r=-0.01, CI=  -6.79 –5.70 | b=-8.54, p=0.12, r=-0.12, CI=  -19.38 –2.29 | b=-1.12, p=0.72, r=-0.03, CI=  -7.29 –5.05 | **b=-12.42, p=0.03, r=-0.16, CI=**  **-23.91 –**  **-0.93** | b=-2.70, p=0.48, r=-0.05, CI=  -10.22 –4.83 | b=4.54, p=0.36, r=0.07, CI=  -5.18 –14.26 | b=-7.41, p=0.32, r=-0.08, CI=  -22.01 –7.20 | b=-11.77, p=0.44, r=-0.06, CI=  -41.62 –18.09 | b=-1.80, p=0.66, r=-0.03, CI=  -9.75 –6.15 | b=1.20, p=0.79, r=0.02, CI=  -7.51 –  9.90 | b=-12.96, p=0.13, r=-0.12, CI=  -29.80 –3.88 | b=0.92, p=0.93, r=0.01, CI=  -21.03 –22.87 | b=-5.98, p=0.18, r=-0.10, CI=  -14.69 –2.72 | b=3.77, p=0.07, r=0.14, CI=  -0.25 –  7.78 | b=-8.26, p=0.36, r=-0.07, CI=  -26.00 –9.47 | b=-2.26, p=0.55, r=-0.05, CI=  -9.73 –  5.21 | b=-4.22, p=0.29, r=-0.08, CI=  -12.06 –3.61 | b=3.65, p=0.06, r=0.14, CI=  -0.19 –7.48 | b=-7.13, p=0.32, r=-0.07, CI=  -21.34 –7.08 | b=-5.67, p=0.12, r=-0.12, CI=  -12.87 –1.54 |
| *SCA*  *(n=106)* | b=2.14, p=0.51, r=0.06, CI=  -4.24 –8.52 | b=-12.71, p=0.03, r=-0.20, CI=  -24.21 –  -1.20 | b=-0.30, p=0.92, r=-0.01, CI=  -6.52-5.91 | **b=-11.75, p=0.04, r=-0.19, CI=**  **-22.91 –**  **-0.60** | b=0.00, p=1.00, r=0.00, CI=  -7.55 –  7.56 | b=4.57, p=0.35, r=0.09, CI=  -5.11 –14.24 | b=-12.32, p=0.15, r=-0.13, CI=  -29.26 –4.62 | b=-9.11, p=0.56, r=-0.05, CI=  -40.19 –21.98 | b=-0.67, p=0.87, r=-0.01, CI=  -8.88 –7.55 | b=0.60, p=0.89, r=0.01, CI=  -8.08 –  9.27 | b=-11.77, p=0.16, r=-0.13, CI=  -28.23 –4.70 | b=0.02, p=1.00, r=0.00, CI=  -21.30-21.34 | b=-4.16, p=0.33, r=-0.09, CI=  -12.64 –4.32 | **b=4.40, p=0.03, r=0.20, CI=**  **0.44** –  **8.35** | b=-18.96, p=0.07, r=-0.17, CI=  -39.13 –1.22 | b=0.53, p=0.89, r=0.01, CI=  -7.16 –  8.21 | b=-4.35, p=0.28, r=-0.10, CI=  -12.27 –3.56 | **b=4.31, p=0.03, r=0.20, CI=**  **0.47** –  **8.16** | b=-5.18, p=0.46, r=-0.07, CI=  -18.99 –8.62 | b=-6.70, p=0.06, r=-0.17, CI=  -13.77 –0.38 |

**Supplementary Table 1. Cont.**

| **Regional Models** | **1a) Lobar SCI** | | **1b) Regional SCI** | | **2a) Lobar SCI + Lobar SCI Number** | | | | **2b) Regional SCI + Regional SCI Number** | | | | **3a) Lobar SCI + Lobar SCI Volume** | | | | **3b) Lobar SCI + Lobar SCI Volume** | | | |
| --- | --- | --- | --- | --- | --- | --- | --- | --- | --- | --- | --- | --- | --- | --- | --- | --- | --- | --- | --- | --- |
| *Predictor* | **SCI Fr.** | **SCI**  **Pa.** | **SCI**  **Dp.** | **SCI**  **Jc.** | **SCI**  **Fr.** | **SCI Fr.**  **No.** | **SCI**  **Pa.** | **SCI Pa. No.** | **SCI Dp.** | **SCI Dp. No.** | **SCI Jc.** | **SCI Jc. No.** | **SCI**  **Fr.** | **SCI Fr.**  **Vol.** | **SCI**  **Pa.** | **SCI Pa. Vol.** | **SCI Dp.** | **SCI Dp. Vol.** | **SCI Jc.** | **SCI Jc. Vol.** |
| **CI** |  |  |  |  |  |  |  |  |  |  |  |  |  |  |  |  |  |  |  |  |
| ***L-SCI***  *all*  *(n=154)* | OR=1.37, p=0.68, CI=  0.31 –  6.05 | OR=0.81, p=0.83,  CI=  0.13 –  5.12 | OR=1.50, p=0.58, CI=  0.36 –  6.26 | OR=0.53, p=0.50, CI=  0.08 –  3.48 | – | OR=1.05, p=0.94, CI=  0.35 –  3.12 | – | OR=1.15, p=0.86, CI=  0.25 –  5.35 | OR=0.93, p=0.94, CI=  0.13 –  6.78 | OR=1.60, p=0.45, CI=  0.47 –  5.44 | OR=0.71, p=0.84, CI=  0.02 –  20.47 | OR=0.66, p=0.77, CI=  0.04 –  10.63 | – | OR=1.01, p=0.95, CI=  0.70 –  1.45 | – | OR=1.07, p=0.73, CI=  0.72 –  1.59 | – | OR=1.06, p=0.78,  CI=  0.69 –  1.64 |  | OR=1.05, p=0.85, CI=  0.62-  1.79 |
| *SCA (n=106)* | OR=0.75, p=0.74, CI=  0.14 –  3.97 | OR=0.81, p=0.84, CI=  0.10 –  6.49 | OR=0.87, p=0.87, CI=  0.17 –  4.44 | OR=0.51, p=0.53, CI=  0.06 –  4.23 | – | OR=0.88, p=0.83, CI=  0.28 –  2.77 | – | OR=1.17, p=0.86, CI=  0.21 –  6.45 | OR=0.45, p=0.49, CI=  0.05 –  4.41 | OR=1.85, p=0.37, CI=  0.49 –  7.09 | OR=0.61, p=0.79, CI=  0.02 –  22.97 | OR=0.81, p=0.89, CI=  0.04 –  15.54 | – | OR=0.88, p=0.52, CI=  0.59 –  1.30 | – | OR=1.14, p=0.55, CI=  0.74 –  1.78 | – | OR=0.96, p=0.88, CI=  0.59-  1.58 |  | OR=1.10, p=0.75, CI=  0.62-  1.96 |
| ***T-SCI***  *all*  *(n=154)* | OR=1.49, p=0.60, CI=  0.33 –  6.72 | OR=0.88, p=0.89, CI=  0.14 –  5.63 | OR=1.66, p=0.51, CI=  0.37 –  7.39 | OR=0.63, p=0.65, CI=  0.09 –  4.44 | – | OR=0.78, p=0.71, CI=  0.21 –  2.87 | – | OR=2.32, p=0.40, CI=  0.33 –  16.45 | OR=1.13, p=0.90, CI=  0.18 –  6.95 | OR=1.57, p=0.44, CI=  0.50 –  4.98 | OR=1.62, p=0.74, CI=  0.09 –  29.19 | OR=0.28, p=0.39, CI=  0.01 –  5.22 | – | OR=0.99, p=0.97, CI=  0.69 –  1.43 | – | OR=1.12, p=0.58, CI=  0.75 –  1.69 | – | OR=1.11, p=0.65, CI=  0.70-  1.77 |  | OR=0.99, p=0.98, CI=  0.57-  1.75 |
| *SCA*  *(n=106)* | OR=0.79, p=0.78, CI=  0.15 –  4.15 | OR=0.90, p=0.92, CI=  0.11 –  7.01 | OR=0.92, p=0.92, CI=  0.18 –  4.79 | OR=0.54, p=0.57, CI=  0.07 –  4.35 | – | OR=0.73, p=0.64, CI=  0.19 –  2.76 | – | OR=2.06, p=0.48, CI=  0.28 –  15.12 | OR=0.53, p=0.54, CI=  0.07 –  4.05 | OR=1.85, p=0.33, CI=  0.54 –  6.40 | OR=1.33, p=0.86, CI=  0.06 –  30.93 | OR=0.28, p=0.46, CI=  0.01 –  7.91 | – | OR=0.87, p=0.50, CI=  0.58 –  1.30 | – | OR=1.19, p=0.45, CI=  0.76 –  1.85 | – | OR=1.05, p=0.84, CI=  0.65-  1.69 |  | OR=0.97, p=0.93, CI=  0.55-  1.72 |
| ***R-SCI***  all  (n=154) | OR=1.38, p=0.68, CI=  0.29 –  6.50 | OR=3.92, p=0.30, CI=  0.30 –  51.99 | OR=2.72, p=0.18, CI=  0.62 –  11.85 | OR=0.00, p=0.99, CI=  0.00 –  inf | – | – | – | – | OR=3.29, p=0.19, CI=  0.55 –  19.61 | OR=0.67, p=0.72, CI=  0.07 –  6.02 | OR=0.00, p=1.00, CI=  0.00 –  inf | OR=0.21, p=1.00, CI=  0.00 –  inf | – | OR=0.24, p=0.30, CI=  0.02 –  3.51 | – | OR=3.81, p=0.15, CI=  0.61 –  23.89 | OR=5.47, p=0.09, CI=  0.77-  39.03 | OR=0.16, p=0.11, CI=  0.02-  1.52 | OR=0.00 p=0.99, CI=  0.00-  inf | OR=9.67p=0.15, CI=  0.43-217.76 |
| *SCA*  *(n=106)* | OR=0.57, p=0.54, CI=  0.10 –  3.41 | OR=9.00, p=0.17, CI=  0.38 –214.21 | OR=1.45, p=0.66, CI=  0.28 –  7.36 | OR=0.00, p=0.99, CI=  0.00 –  inf | – | – | – | – | – | OR=1.13, p=0.90, CI=  0.16 –  7.90 | – | OR=0.00, p=0.99, CI=  0.00 –  inf | – | OR=0.04, p=0.08, CI=  0.00 –  1.46 | – | OR=10.90, p=0.05, CI=  1.02 –116.22 | – | OR=0.33, p=0.21, CI=0.06-1.83 |  | OR=3.57, p=0.25, CI=0.41-31.35 |

**Supplementary Table 1. Cont.**

| **Regional Models** | **4) No of SCI Zones** | **4) No of SCI Zones** | **4) No of SCI Zones** | **4) No of SCI Zones** |
| --- | --- | --- | --- | --- |
| *Outcome* | **FSIQ** | **WMI** | **PSI** | **CI** |
| *Predictor* |  |  |  |  |
| ***L-SCI***  *all (n=154)* | b=-0.39, p=0.62, r=-0.04, CI=-1.93 – 1.15 | b=-0.45, p=0.60, r=-0.04,  CI=-2.13 – 1.23 | b=-0.62, p=0.43, r=-0.06,  CI=-2.15 – 0.92 | OR=1.00, p=1.00,  CI=0.64 – 1.56 |
| *SCA (n=106)* | b=-0.19, p=0.83, r=-0.02, CI=-1.96 – 1.58 | b=-0.23, p=0.81, r=-0.02,  CI=-2.16 – 1.69 | b=-0.27, p=0.75, r=-0.03,  CI=-1.97 – 1.42 | OR=0.90, p=0.69,  CI=0.54 – 1.51 |
| ***T-SCI***  *all (n=154)* | b=-0.12, p=0.89, r=-0.01, CI=-1.87 – 1.63 | b=-0.12, p=0.90, r=-0.01,  CI=-2.03 – 1.80 | b=-0.64, p=0.47, r=-0.06,  CI=-2.39 – 1.11 | OR=1.08, p=0.74,  CI=0.69 – 1.69 |
| *SCA (n=106)* | b=0.08, p=0.93, r=0.01,  CI=-1.87 – 2.04 | b=-0.04, p=0.97, r=-0.00,  CI=-2.16 – 2.09 | b=-0.30, p=0.76, r=-0.03,  CI=-2.17 – 1.58 | OR=0.98, p=0.94,  CI=0.57 – 1.67 |
| ***R-SCI***  all (n=154) | b=-0.11, p=0.96, r=-0.00, CI=-3.94 – 3.73 | b=-1.37, p=0.52, r=-0.05,  CI=-5.56 – 2.83 | b=-2.53, p=0.19, r=-0.10, CI=-6.34 – 1.29 | OR=1.52, p=0.41,  CI=0.56 – 4.11 |
| *SCA (n=106)* | b=-0.11, p=0.95, r=-0.01,  CI=-4.12 – 3.89 | b=-1.38, p=0.53, r=-0.06,  CI=-5.73 – 2.97 | b=-2.22, p=0.25, r=-0.11, CI=-6.04 – 1.60 | OR=1.13, p=0.84,  CI=0.35 – 3.58 |

**Supplementary Table 1. Cont.**

| Study details | | | SCA (N) | SCA  (age) | C (N) | C  (age) | SCI definition | MRI | Sequence Res. | Lesion Metric | % SCA w/ SCI | %C  w/ SCI | Cognitive Tests | Neg. effect of SCI - FSIQ, WMI, or PSI | Neg. effect of SCI - any domain | Results: Assoc w/cognition or other neuroimaging outcome |
| --- | --- | --- | --- | --- | --- | --- | --- | --- | --- | --- | --- | --- | --- | --- | --- | --- |
|  | | | | | | | | | | | | | | | | |
| Armstrong | US | 1996 | 135 | 6-12y | - | - | area of abnormally increased signal on T2 | - | *Not reported* | Prevalence (i.e. binary y/n) | 16% | - | WISC-III (composite scores) | Y | Y | SCI+ significantly lower VIQ (79.9 vs 88.8) and FSIQ (82.8 vs 90.0) |
| Bernaudin et al | FR | 2000 | 121 | 5-15y | NA* | NA* | L-SCI: area of abnormally increased signal on T2 and PD | 1T, 1.5T | *Not reported* | Prevalence (i.e. binary y/n) | 14% | - | WISC-III (full scores) | N | Y | No differences between SCI+ vs SCI- in FSIQ, PIQ, PS, but SCI+ group significantly reduced verbal comprehension scores (82.1 vs. 96.4) |
| Wang et al *follow-up*  *from*  *Armstrong* | US | 2000 | 78 | 6-16y | - | - | T-SCI: area of abnormally increased signal T2 and FLAIR - min 3mm | 0.6, 1.0, 1.5T | *Not reported* | Prevalence (i.e. binary y/n) | 22% | - | WISC-III (composite scores) | Y | Y | SCI+ group significantly reduced FSIQ (73 vs 86), PIQ (79.5 vs 87.7), and VIQ (70.9 vs 86.8) |
| Brown et al | US | 2000 | 63 | 6-17y | - | - | *No explicit definition* | *-* | *Not reported* | Prevalence (i.e. binary y/n) | 17% |  | Large battery across domains, incl WISC-II (composite scores) | N | Y | No difference between overt stroke, SCI+ and SCI-in FSIQ, VIQ, PIQ, reading, or math (WJR), SCI+ group took significantly longer to complete the trail making test of executive function than the overt stroke and SCI- group |
| Wang et al | US | 2001 | 185 | 6-12y | - | - | SCI: area of abnormally increased signal on T2 and PD | 0.6, 1.0, 1.5T | *Not reported* | Prevalence (i.e. binary y/n) | 17% | - | WISC-R/III (composite scores) | Y | Y | SCI+ group significantly reduced FSIQ (77.2 vs 84.8), VIQ (77.1 vs 85.3), no differences in PIQ |
| Schatz et al | US | 2001 | 64 | 8-12y | NA* | NA* | T-SCI: area of abnormally increased signal T2 and PD - min 3mm | 1.5T | 5mm slice thickness, 2mm slice gap - no further details | Prevalence (i.e. binary y/n) | 30%  (NR*) | - | Comprehensive battery across domains | - | Y | SCI+ group showed a higher rate of deficits (1.5 SD below norm) overall than SCI- group (79% vs 36%) |
| Schatz et al | US | 2002 | 27 | M  SCI+=  12.4, SD=1.9, M SCI-=11.6, SD=3.0 | - | - | T-SCI: area of abnormally increased signal T2 and PD - min 3mm | 1.5T | 5mm slice thickness, 2mm slice gap - no further details | Volume (groups: SCI-, SCI+ low, SCI+ high) | 67% (NR*) | - | WASI (composite scores) | Y | Y | SCI+ high volume group significantly reduced FSIQ compared to SCI+ low volume and SCI- groups (76.1, 87.7, 89.9), no between group differences in VIQ or PIQ |
| Thompson et al | US | 2003 | 127 (visit 1) | 5-15y | - | - | L-SCI: area of abnormally increased signal on T2 and PD | 0.6, 1, or 1.5T | *Not reported* | Prevalence (i.e. binary y/n) | 23% | - | WISC-R/III (composite scores), CBC | N | Y | No significant differences between SCI+ and SCI- groups in FSIQ, PIQ or behavioural problems, but significantly reduced VIQ in SCI+ group (84.6 vs 93.0) |
| Steen et al | US | 2003 | 49 | 4-19y | - | - | L-SCI: any lesion was sufficient to be classified as abnormal | 1.5T | *Not reported* | Prevalence (i.e. binary y/n) | 43%  (NO*) |  | WISC-R/III (composite scores) | N | Y | Abnormal imaging group showed reduced VIQ (74.1 vs 84.6), and VC factor (74.4 vs 86.0), but no differences in FSIQ, PIQ, PO, FD, or PS |
| Grueneich et al | US | 2004 | 31 | 9-16y | - | - | L-SCI: structural features scored on T1 and T2, Perfusion studies scored | 1.5T | *Not reported* | Prevalence (i.e. binary y/n) | 45% (NO*) | - | Large battery across domains, incl WISC-R | N | N | Neuroimaging abnormalities were not significantly correlated with level of neuropsychological performance |
| Zafeiriou et al | GR | 2004 | 11 | 7-20y | - | - | L-SCI: area of abnormally increased signal on T2 | 1.5T | *Not reported* | Prevalence (i.e. binary y/n) | 38% | - | WISC-III (composite scores) | N | N | No differences between SCI+ and SCI- groups in FSIQ, VIQ, or PIQ |
| Steen et al | US | 2005 | 54 | M=10.9, SD=2.9 | NA* | NA* | L-SCI: focal brain injury of any type | 1.5T | 3-5mm slice thickness, 1-3mm slice gap - no further details | Prevalence (i.e. binary y/n) | 44%  (NO*) | - | WISC-III (composite scores) | N | N | No significant differences between patients normal and abnormal by MRI in FSIQ, VIQ, PIQ, VCI, PO, PS |
| Baldeweg et al | UK | 2006 | 36 | 9-24y | 31 | 8-25y | L-SCI: area of abnormally increased signal on T2 and PD | 1.5T | *Not reported* | Prevalence (i.e. binary y/n) | 44% | 0% | WISC-III | Y | Y | SCI+ group significantly lower VIQ (84 vs 93), FSIQ and PIQ scores (both 82 vs 92). |
| Melek et al | TK | 2006 | 59 | 16-35y | NA* | NA* | *No explicit definition* | 1.5T | 5mm slice thickness - no further details | Prevalence (i.e. binary y/n) | 36% | - | Neurological soft signs (NSS) | - | Y | SCI+ group had significantly higher NSS scores than SCI- group (4.04 vs 1.57) |
| Hogan et al | UK | 2006 | 30 | M=17.4, SD=4.2 |  |  | *No explicit definition* | 1.5T | *Not reported* | Prevalence (i.e. binary y/n) | 57% (*NR) | - | WISC-III/R (FSIQ only) | N | N | No difference in FSIQ between SCI+ and SCI- groups |
| Kral et al | US | 2006 | 27 | 6-16y |  |  | L-SCI: area of abnormally increased signal on T2 and PD | *-* | *Not reported* | Prevalence (i.e. binary y/n) | 19% |  | Large battery across domains, incl WASI (IQ only) | N | Y | Presence of SCI positively associated with working memory, and negatively associated with cognitive flexibility in univariate analyses. Not retained as a predictor in step-wise analyses |
| Schatz et al | US | 2006 | 28 | M SCI+= 12.2, SD=1.2, M SCI-=12.3, SD=2.7 | NA* | NA* | T-SCI: area of abnormally increased signal T2 - min 3mm | 1.5T | 5 mm slice thickness, 2mm slice gap - no further details | Prevalence (i.e. binary y/n), Volume - but included overt stroke | 29% |  | WISC-III, Self-ordered pointing test | N | Y | No differences between SCI- and SCI+ groups on FSIQ, VCI, PO, or PS, but SCI+ group had a higher rate of errors on the pointing test, lesion volume significantly predicted scores across domains (however; included overt stroke) |
| White et al | US | 2006 | 65 | 6-16y | - | - | R-SCI: presence of infarction on T1 + T2 (no minimal length specified) | *-* | *Not reported* | Prevalence (i.e. binary y/n) | 25% | - | WASI, WIAT, CLVT, CMS | N | Y | No differences between groups in WASI or CMS scores, but significantly reduced WIA math reasoning (39.3 vs 45.0), CVLT learning slope (44.1 vs 51.7), and CMS numbers backward (41.0 vs 47.6) and increased CVLT preservations (50.2 vs 55.6) in SCI+ group |
| Hogan et al  *follow-up* | UK | 2008 | 36 | *Not reported* | 15 | 17.5 (2.1) | *No explicit definition* | *-* | *Not reported* | Prevalence (i.e. binary y/n) | 50%  (NR*) |  | WISC-III/R | N | N | No evidence of decline in VIQ or PIQ in SCI+ or SCI- groups between childhood and adolescence |
| Gold et al | US | 2008 | 65 | 7-17y | - | - | *No explicit definition* | *-* | *Not reported* | Prevalence (i.e. binary y/n) | 13% | *-* | WISC-R/III (composite scores) | N | N | No differences between SCI+ and SCI- groups in VIQ or PIQ |
| Wang et al | US | 2008 | 23 | 0-1y *(10-18 months)* | - | - | L-SCI: area of abnormally increased signal on FLAIR | *-* | 5 mm slice thickness, minimal slice gap | Prevalence (i.e. binary y/n) | 13% | - | BSID-II | N | N | SCI were not associated with mental developmental index score |
| Vichinsky et al | US | 2010 | 141 | M=31.6,  SD=  8.95 | 44 | M=  33.1,  SD=  10.06 | R-SCI: increased signal T2, decreased signal on T1 - min 5mm | 1.5T | *Not reported* | Prevalence (i.e. binary y/n) | 13% | 2% | WAIS-II (composite scores), D-KEFS, TEA | N | N | No differences as a function of SCI were observed |
| Montanaro et al | Italy | 2013 | 68 | 4-15y | - | - | T-SCI: area of abnormally increased signal T2 and FLAIR - min 3mm | 1.5T | 5mm slice thickness, 0.5mm gap (no further details) | Volume (groups: SCI-, SCI+ low, SCI+ high) | 36% | - | WPSSI, WISC-III (composite scores) | N | N | No differences as a function of SCI group detected on FSIQ, VIQ, or PIQ |
| Smith et al | US | 2013 | 82 | 6-12y | - | - | *No explicit definition* | *-* | *Not reported* | Prevalence (i.e. binary y/n) | 16%  (NO*) | - | - | - | - | Stroke status (overt or silent) not a significant independent predictor of academic achievement |
| Mackin et al | US | 2014 | 120 | M=32.0,  SD=9.0 | 33 | M= 34.4  SD= 10.3 | R-SCI: area of abnormally increased T2 + PD + decreased T1 - min 5mm | 1.5T | *Not reported* | Prevalence (i.e. binary y/n) | 15% | 3% | - | - | - | Effect of SCI not examined/reported |
| King et al *SIT baseline (B/L)* | US | 2014 | 150 | 5-15y | - | - | T-SCI: area of abnormally increased signal T2 and FLAIR - min 3mm | 1, 1.5T | 5 mm slice thickness, 0-1mm slice gap - no further details | Prevalence (i.e. binary y/n) | 71%* (NR*) | - | WASI (IQ only) | Y | Y | B/L SCI presence associated with a 5 point decrease in IQ scores in multivariate models. |
| DeBaun et al  *SIT RCT* | US, Eur, Can | 2014 | 196 | 5-15y | - | - | T-SCI: area of abnormally increased signal T2 and FLAIR - min 3mm | 1, 1.5T | 5 mm slice thickness, 0-1mm slice gap - no further details | Prevalence (i.e. binary y/n) | 100%  (NR*) | - | WASI | - | - | SIT RCT: no significant change over 3 y in PIQ, VIQ, FSIQ or BRIEF between transfusion and standard care |
| Smith et al | US | 2016 | 32 | 8-17y | NA* | NA* | T-SCI: area of abnormally increased signal T2 and PD - min 3mm | 1.5T | *Not reported* | Prevalence (i.e. binary y/n), Volume but included overt stroke | 47% (*NR) |  | Examiner, WJ-II | - | Y | Frontal-parietal lesion volume negatively correlated with WMI, cognitive control, VC, and WJ numbers reversed. Sub-cortical lesion volume negatively correlated with WMI, PS, VC and visual matching (included overt stroke) |
| Choudhury et al | US | 2018 | 150 | 5-14y | - | - | T-SCI: area of abnormally increased signal T2 and FLAIR - min 3mm, R-SCI: increased T2 and FLAIR, and decreased T1 - min 5mm | 1, 1.5T | 5 mm slice thickness, 0-1mm slice gap - no further details | Prevalence (i.e. binary y/n) | T-SCI:  100%*  R-SCI: 27%* (NR*) | - | WASI (IQ only) | N | Y | T-SCI presence associated with a 5 point decrease in IQ scores in multivariate models, but no effect of R-SCI presence |
| Jacob et al | TZ | 2022 | 73 | 6-16y | 71 | 6-16y | T-SCI: area of abnormally increased signal T2 and FLAIR - min 3mm | 1.5T | 5 mm slice thickness | Prevalence (i.e. binary y/n) | 30% | 5% | WISC  PSI, WMI, PRI  Raven’s | N | N | ANOVA: significant differences between SCD and controls for PSI but not for WMI, PRI, Raven’s. Although SCI+ lower than SCI- none significantly different |
| Supplementary Table 2. Overview of 1.5T MRI studies examining impact of SCI on cognitive outcomes in patients with SCD. Note: there may be between-study overlap in participants. Abbreviations: SCD=Sickle Cell Disease, C=Control, N=no; Y=yes; NA*=controls included for cognitive assessment but MRI not assessed/reported), NR*=not representative (e.g. participants selected for presence of SCI), NO*=not only SCI included in definition of abnormality, WPPSI/WISC/WAIS=Wechsler intelligence scale for preschool/children/adults, WASI = Wechsler abbreviated scale of intelligence, CBC = child behaviour checklist, WIAT = Wechsler Individual Achievement Test, CVLT=California Verbal Learning Test, CMS = Children’s memory scale, BSID-II=Bayley Scales of Infant Development, FSIQ, VIQ, PIQ, PSI=processing speed, WM=working memory; VC=verbal comprehension PO=perceptual organisation, FD=freedom from distractibility; WJ= Woodcock Johnson Test of Cog Ability; D-KEFS=Delis-Kaplan executive function system, TEA=Test of Everyday Attention, Raven’s=Raven’s progressive matrices; NSS=Neurological soft signs; Res=Resolution; PD=Proton Density; FLAIR= fluid attenuated inversion recovery; L-/T-/R-SCI = Liberal/Traditional/Restrictive silent cerebral infarction definitions; ANOVA=Analysis of Variance, M=Mean, SD=standard deviation; CSSCD=Co-operative study of sickle cell disease; SIT=Silent infarct Transfusion trial, B/L baseline, RCT=randomised controlled trial; US=United States, Eur=Europe, Can=Canada, UK=United Kingdom, FR=France, TK=Turkey, GR=Greece, TZ=Tanzania | | | | | | | | | | | | | | | | |

**References for Supplementary Table 2**

Armstrong FD, Thompson RJ Jr, Wang W, et al. Cognitive functioning and brain magnetic resonance imaging in children with sickle Cell disease. Neuropsychology Committee of the Cooperative Study of Sickle Cell Disease. Pediatrics. 1996;97:864-70.

Baldeweg T, Hogan AM, Saunders DE, et al. Detecting white matter injury in sickle cell disease using voxel-based morphometry. Ann Neurol. 2006;59:662–672.

Bernaudin F, Verlhac S, Fréard F, et al. Multicenter prospective study of children with sickle cell disease: radiographic and psychometric correlation. J Child Neurol. 2000;15:333–343.

Brown RT, Davis PC, Lambert R, Hsu L, Hopkins K, Eckman J. Neurocognitive Functioning and Magnetic Resonance Imaging in Children With Sickle Cell Disease. J Pediatr Psychol. 2000;25:503–513.

DeBaun MR, Gordon M, McKinstry RC, et al. Controlled trial of transfusions for silent cerebral infarcts in sickle cell anemia. N Engl J Med. 2014;371(8):699-710.

Choudhury, N. A., DeBaun, M. R., Rodeghier, M., King, A. A., Strouse, J. J., and McKinstry, R. C. Silent cerebral infarct definitions and full-scale IQ loss in children with sickle cell anemia. Neurology 2018;90, e239–e246.

Hogan AM, Pit-ten Cate IM, Vargha-Khadem F, Prengler M, Kirkham FJ. Physiological correlates of intellectual function in children with sickle cell disease: hypoxaemia, hyperaemia and brain infarction. Dev Sci. 2006;9:379–387.

Hogan AM, Telfer P, Prengler M, Saunders D, Wade AM, Vargha-Khadem F, Kirkham FJ. Intellectual function in children with sickle cell anemia: Longitudinal data from the East London cohort. Br J Haem 2008; 141 suppl 1: 111 (301).

Gold JI, Johnson CB, Treadwell MJ, Hans N, Vichinsky E. Detection and assessment of stroke in patients with sickle cell disease: neuropsychological functioning and magnetic resonance imaging. Pediatr Hematol Oncol. 2008;25:409–421.

Grueneich R, Ris MD, Ball W, et al. Relationship of structural magnetic resonance imaging, magnetic resonance perfusion, and other disease factors to neuropsychological outcome in sickle cell disease. J Pediatr Psychol. 2004;29:83–92.

Jacob M, Stotesbury H, Kija E, Saunders D, Mtei RJ, Tutuba H, Masanu U, Kilonzo M, Kazema R, Hood AM, Kirkham F, Dimitriou D, Makani J. Effect of age, cerebral infarcts, vasculopathy and haemoglobin on cognitive function, in Tanzanian children with sickle cell anaemia. Eur J Paediatr Neurol. 2022 Mar;37:105-113.

King AA, Strouse JJ, Rodeghier MJ, et al. Parent education and biologic factors influence on cognition in sickle cell anemia. Am J Hematol. 2014;89:162–167.

Kral MC, Brown RT, Connelly M, et al. Radiographic Predictors of Neurocognitive Functioning in Pediatric Sickle Cell Disease. J Child Neurol. 2006;21:37–44.

Mackin RS, Insel P, Truran D, et al. Neuroimaging abnormalities in adults with sickle cell anemia: associations with cognition. Neurology. 2014;82:835–841.

Melek I, Akgul F, Duman T, Yalcin F, Gali E. Neurological soft signs as the stroke risk in sickle cell disease. Tohoku J Exp Med. 2006;209:135–140.

Montanaro M, Colombatti R, Pugliese M, et al. Intellectual function evaluation of first generation immigrant children with sickle cell disease: the role of language and sociodemographic factors. Ital J Pediatr. 2013;39:36.

Schatz J, Brown RT, Pascual JM, Hsu L, DeBaun MR. Poor school and cognitive functioning with silent cerebral infarcts and sickle cell disease. Neurology. 2001;56:1109–1111.

Schatz J, White DA, Moinuddin A, Armstrong M, DeBaun MR. Lesion burden and cognitive morbidity in children with sickle cell disease. J Child Neurol. 2002;17:891–895.

Schatz J, Buzan R. Decreased corpus callosum size in sickle cell disease: relationship with cerebral infarcts and cognitive functioning. J Int Neuropsychol Soc. 2006;12:24–33.

Smith KE, Patterson CA, Szabo MM, Tarazi RA, Barakat LP. Predictors of Academic Achievement for School Age Children with Sickle Cell Disease. Adv Sch Ment Health Promot. 2013;6:5–20.

Smith KE, Schatz J. Working Memory in Children With Neurocognitive Effects From Sickle Cell Disease: Contributions of the Central Executive and Processing Speed. Dev Neuropsychol. 2016;41(4):231-244.

Steen RG, Miles MA, Helton KJ, et al. Cognitive impairment in children with hemoglobin SS sickle cell disease: relationship to MR imaging findings and hematocrit. AJNR Am J Neuroradiol. 2003;24:382–389.

Steen RG, Fineberg-Buchner C, Hankins G, Weiss L, Prifitera A, Mulhern RK. Cognitive Deficits in Children With Sickle Cell Disease. J Child Neurol. 2005;20:102–107.

Thompson RJ, Armstrong FD, Link CL, Pegelow CH, Moser F, Wang WC. A prospective study of the relationship over time of behavior problems, intellectual functioning, and family functioning in children with sickle cell disease: a report from the Cooperative Study of Sickle Cell Disease. J Pediatr Psychol. 2003;28:59–65.

Vichinsky EP, Neumayr LD, Gold JI, et al. Neuropsychological dysfunction and neuroimaging abnormalities in neurologically intact adults with sickle cell anemia. JAMA. 2010;303:1823–1831.

Wang WC, Gallagher DM, Pegelow CH, et al. Multicenter comparison of magnetic resonance imaging and transcranial Doppler ultrasonography in the evaluation of the central nervous system in children with sickle cell disease. J Pediatr Hematol Oncol. 2000;22:335–339.

Wang W, Enos L, Gallagher D, et al. Neuropsychologic performance in school-aged children with sickle cell disease: a report from the Cooperative Study of Sickle Cell Disease. J Pediatr. 2001;139:391–397.

Wang WC, Pavlakis SG, Helton KJ, et al. MRI abnormalities of the brain in one-year-old children with sickle cell anemia. Pediatr Blood Cancer. 2008;51:643–646.

White DA, Moinuddin A, McKinstry RC, Noetzel M, Armstrong M, DeBaun M. Cognitive screening for silent cerebral infarction in children with sickle cell disease. J Pediatr Hematol Oncol. 2006;28:166–169.

Zafeiriou DI, Prengler M, Gombakis N, et al. Central nervous system abnormalities in asymptomatic young patients with Sb-thalassemia. Ann Neurol. 2004;55:835–839.

**Supplementary figure 1. Correlations between predictors for models run in people with SCA only.** Showing relationships between continuous variables included in the global regression models run in patients with sickle cell anemia only. Values are zero-order Spearman’s rank correlation coefficients. Shaded areas represent significant relationships (i.e. p <0.05), with blue used to represent negative relationships, orange used to represent positive relationships, and colour intensity used to represent the strength of the relationships. Abbreviations: ICV = intra-cranial volume, Edu. Decile = education decile; Hb = hemoglobin; SpO_2_ = peripheral oxygen saturation; L-/T-/R-SCI = Liberal/Traditional/Restrictive silent cerebral infarction definitions; No. = Number; Vol = Volume; FSIQ = full-scale IQ; WMI = working memory index; PSI = processing speed index

**Supplementary Figure 2. Lesion distribution for traditionally-defined SCI.** Visualising the distribution of lesions for the traditional definition (T-SCI) using bullseye plots, where sectors represent the four lobes (F=frontal, P=parietal, O=occipital, T=temporal) and rings represent three regions; periventricular (PV; interior ring; red), deep (DP; middle ring; green), and juxta-cortical (JC; exterior ring; blue) yielding 12 regional lobar zones, with distribution represented across three metrics; the proportion of the total group with lesion voxels in particular zone (left - % of total group), the proportion of the total lesion number classed as belonging to regional-lobar zone (middle - % of total lesion number), and the proportion of the total lesion voxels in particular regional-lobar zone (right - % of total lesion volume).

**Supplementary Figure 3. Lesion distribution for restrictively-defined SCI.** Visualising the distribution of lesions for the restrictive definition (R-SCI) using bullseye plots, where sectors represent the four lobes (F=frontal, P=parietal, O=occipital, T=temporal) and rings represent three regions; periventricular (PV; interior ring; red), deep (DP; middle ring; green), and juxta-cortical (JC; exterior ring; blue) yielding 12 regional lobar zones, with distribution represented across three metrics; the proportion of the total group with lesion voxels in particular zone (left - % of total group), the proportion of the total lesion number classed as belonging to regional-lobar zone (middle - % of total lesion number), and the proportion of the total lesion voxels in particular regional-lobar zone (right - % of total lesion volume).
